# From overlooked to outstanding: a molecular silver complex in heterogeneous CO_2_ electroreduction

**DOI:** 10.1039/d6sc00957c

**Published:** 2026-06-04

**Authors:** Wiebke Wiesner, Kevinjeorjios Pellumbi, Julia Jökel, Ulf-Peter Apfel

**Affiliations:** a Ruhr-Universität Bochum, Fakultät für Chemie und Biochemie, Activation of Small Molecules/Technical Electrochemistry Universitätsstr. 150 44801 Bochum Germany ulf.apfel@rub.de ulf-peter.apfel@umsicht.fraunhofer.de; b Fraunhofer UMSICHT, Department Power-to-Chemicals Osterfelderstr. 3 46047 Oberhausen Germany

## Abstract

Heterogenized molecular transition metal complexes, especially silver-based ones, have shown to be highly efficient as catalysts for electrochemical CO_2_ reduction (eCO_2_R). Herein we present the application of a silver dithiacyclam polymer (dithiacyclam = 1,8-dithia-4,11-diazacyclotetradecane, Ag(dithiacyclam)) in homogeneous as well as heterogeneous eCO_2_R. Although the complex does not exhibit a noteworthy activity in solution, changing the catalyst environment through heterogenization onto a gas diffusion electrode (GDE) and subsequent application in a zero-gap electrolyzer (ZGE) drastically boosts its catalytic performance. At a current density of 50 mA cm^−2^ a remarkable FE_CO_ of 97% is reached. Moreover, we highlight how optimization of the GDE fabrication *via* ink engineering including the choice of dispersion solvent results in a FE_CO_ up to 90% at an elevated current density of 300 mA cm^−2^. Even at more application oriented current densities of 500 mA cm^−2^ the eCO_2_R outcompetes the competing hydrogen evolution reaction, achieving a FE_CO_ of 55%. Although signs of catalyst transformation into silver particles are observed in post-mortem analysis, these particles show higher activity than commercially available silver nanoparticles, thus highlighting that molecular systems can be very promising catalyst precursors for efficient eCO_2_R.

## Abstract 2

A silver cyclam catalyst was tested in electrochemical CO_2_ reduction. Despite its inactivity in homogeneous catalysis, it exhibits excellent performance in a zero-gap electrolyzer with a maximum faradaic efficiency of 90% at 300 mA cm^−2^.

## Graphical abstract

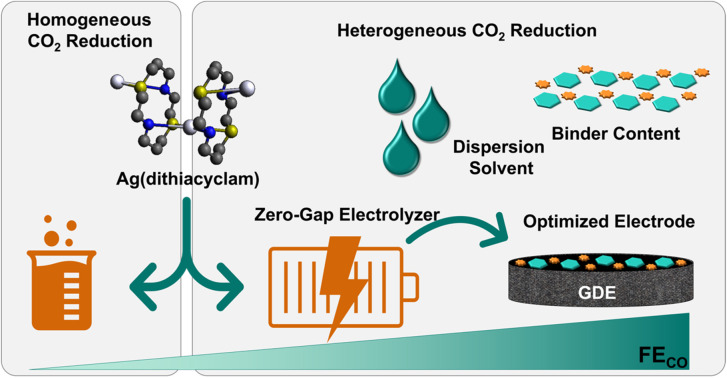

## Introduction

**Fig. 1:**
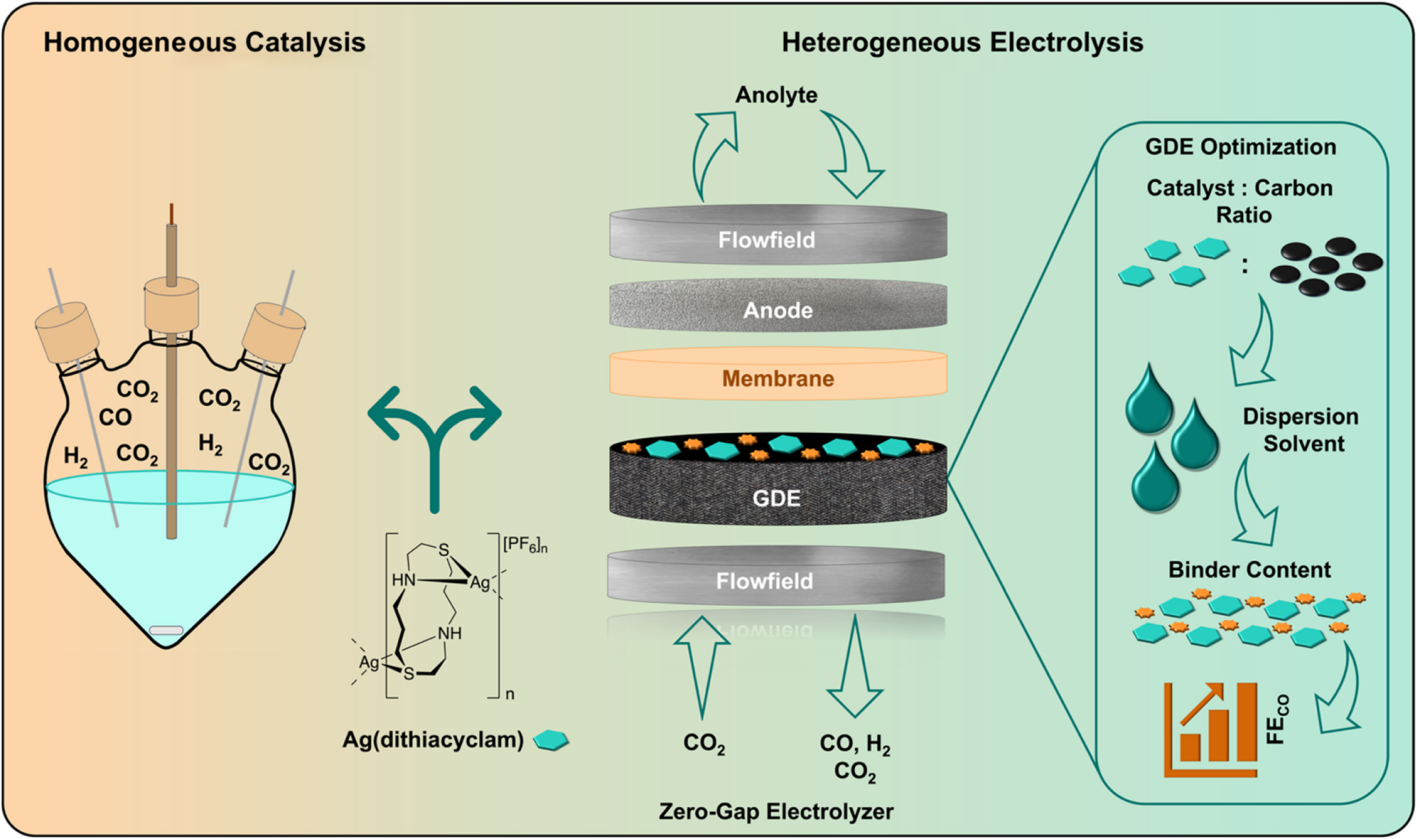
Graphical overview of the herein presented work: tuning the electrolysis environment of Ag(dithiacyclam) from a homogeneous solution to a heterogeneous environment within a GDE along with tailoring of the GDE composition to maximise the eCO_2_R activity.

Electrochemical CO_2_ reduction (eCO_2_R) is a promising approach to sustainably convert the ubiquitous waste product CO_2_ into valuable building blocks such as CO, ethylene and methane for the chemical industry.^1,2^ Depending on the investigation goals – fundamental understanding or application oriented performance – the choice of electrolyzer type plays a crucial role. Batch- or H-type electrolyzers are commonly applied to test intrinsic catalyst properties, either in a dissolved or heterogenized form. However, these systems are not suitable for scale up as they are not designated for continuous-flow operation. In addition, they exhibit several limitations such as high cell resistances caused by the liquid electrolyte, resulting in increased energy consumption, and CO_2_ mass transport limitations arising from its limited solubility in the chosen electrolyte. A scalable zero-gap electrolyzer (ZGE) omits the previously mentioned disadvantages by operating without liquid electrolyte compartments.^3–6^ Furthermore, it has been shown to be able to operate under industrially relevant conditions of high current densities (>200 mA cm^−2^), elevated temperatures (>60 °C) and cell voltages <3 V.^1,7,8^ Commonly a gas diffusion electrode (GDE) is employed as a cathode, in which the catalyst is coated onto a layer of woven carbon fibres, the so called gas diffusion layer (GDL), which is directly supplied with gaseous CO_2_. The catalyst layer (CL) commonly consists of a catalyst, a conductive carbon support and an ionomeric binder. This GDE is directly in contact with the anode *via* an ion-permeable solid polymer membrane, which is commonly an anion exchange membrane (AEM) (Fig. 1).^2,6,9–11^

Previous studies over several decades have demonstrated that transition metal complexes exhibit a high activity for eCO_2_R in homogeneous catalysis.^12–15^ As the research on heterogenized molecular catalysts emerged during recent years, it was found that the high activity achieved in solution is not necessarily transferable onto other electrolyzer types like a ZGE. This behaviour was recently highlighted for iron tetraphenyl porphyrin (FeTPP) which is well known for its homogeneous eCO_2_R activity.^16–21^ Yet, it collapsed once implemented into a ZGE even at low current densities of ≤100 mA cm^−2^.^20–22^ In contrast, molecular silver complexes which are not known to homogeneously catalyse the eCO_2_R achieved remarkable performances when applied in ZGEs.^6,12,22,23^ A good example to showcase the efficiency of silver complexes in ZGEs is silver-based BIAN complexes (BIAN = bis(imino)acenaphthene) which reached a remarkable faradaic efficiency for CO (FE_CO_) of 67% at a current density of 600 mA cm^−2^ after careful optimization of the GDE fabrication.^10^

For the numerous examples of molecular electrocatalysts in eCO_2_R it is well established that the performance is highly dependent on its environment: in homogeneous electrolysis this mainly comprises chosen solvent, catalyst concentration and kind and concentration of proton source.^12,21^ However, immobilized complexes in ZGEs are affected by a variety of different parameters, such as catalyst loading, the nature and the amount of the carbon support, the dispersion medium, or the binder composition, which determine the local catalyst environment in the CL. Besides, process parameters like applied current density, anolyte composition, gas flow or humidification thereof influence the catalysis and performance.^6,10,24,25^

While individual studies have addressed some of the mentioned parameters, mainly in metallic systems, a holistic optimization approach under industrially relevant conditions (≥200 mA cm^−2^, ≥60 °C) for molecular ones remains unexplored.^26,27^ Hence, we sought to present a comprehensive study of a previously untested molecular catalyst for eCO_2_R encompassing both its homogeneous catalytic activity and its performance under application-relevant ZGE conditions. To design this study, we took advantage of the extensive literature on molecular transition metal complexes and selected the previously described Ag(dithiacyclam) complex (dithiacyclam = 1,8-dithia-4,11-diazacyclotetradecane) motivated by three main considerations. First, the cyclam ligand system (1,4,8,11-tetraazacyclotetradecane) is well established, and complexes derived from it have been thoroughly investigated as homogeneous eCO_2_R catalysts due to their high efficiencies in aqueous solutions.^28,29^ However, their use in ZGEs, particularly in combination with commonly applied AEMs, has been rarely explored.^30^ This limited application is largely due to the high aqueous solubility of transition metal complexes of the standard cyclam ligand, which is unsuitable for ZGE catalysis because the catalyst may be washed out of the GDE.^31^ To overcome this limitation, we selected the sulfur-containing cyclam analogue dithiacyclam, as its complexes are typically water-insoluble while remaining active in eCO_2_R.^32,33^ At last, we chose silver as the metal centre because silver-based molecular systems are currently the best-performing catalysts in ZGEs, and the coordination environment of dithiacyclam is well suited to accommodate the soft Ag(i) ion.^22,24,34^ As reported previously, dithiacyclam forms water-insoluble polymeric Ag(dithiacyclam) structures upon coordination with Ag(i), making this complex particularly well suited for the purpose of our study.

Thus, we herein present a study comprising homogeneous and heterogeneous investigations using Ag(dithiacyclam) as a catalyst in eCO_2_R. Our findings underscore not only the importance of probing catalytic systems directly in heterogeneous environments but also how much potential performance may be overlooked when relying solely on homogeneous catalyst screening. Exploring the difference of homogeneous and heterogeneous operation opens a largely untapped field with significant implications for catalyst development and industrial electrochemical processes.

## Results and discussion

### Homogeneous electrochemistry

**Fig. 2:**
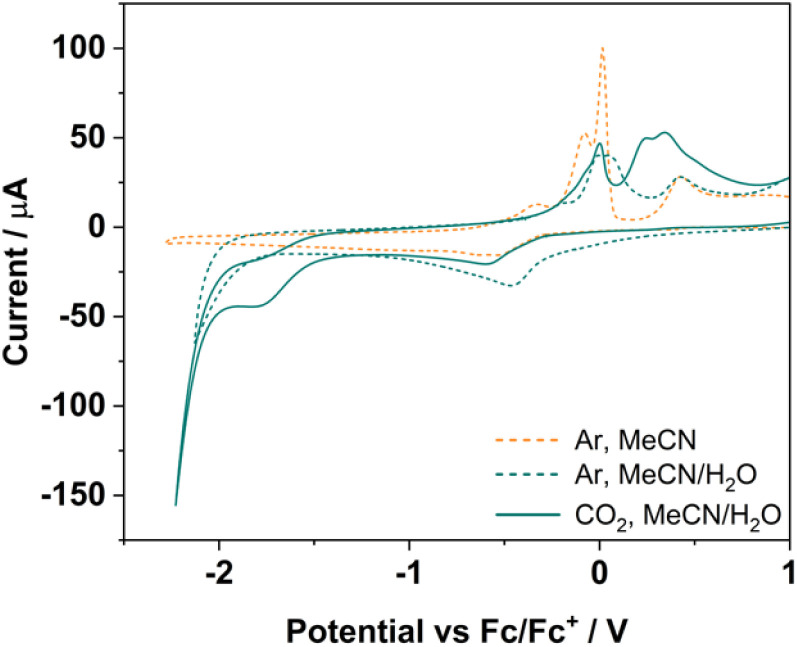
CVs of Ag(dithiacyclam) (0.1 mM) recorded at a scan speed of 100 mV s^−1^ with 0.1 M TBAPF_6_ as supporting electrolyte in pure MeCN in an Ar atmosphere (orange dashed line) or in MeCN/H_2_O 4 : 1 with 0.1 M TBAPF_6_ as supporting electrolyte, either in an Ar (dark green dashed line) or CO_2_ (dark green line) atmosphere.

The herein investigated Ag(dithiacyclam) was prepared according to a literature known procedure.^34^ Electrochemical investigations thereof were performed in a one compartment cell using a glassy carbon (3 mm diameter) working electrode, a platinum wire as a counter electrode and a silver wire as a pseudo reference electrode. Ferrocene (Fc) was added as an internal reference and all cyclic voltammograms (CVs) were referenced against the Fc/Fc^+^ redox couple. CVs of the complex were recorded in a 1 mM acetonitrile (MeCN) or MeCN : H_2_O (4 : 1) mixture either in an inert Ar atmosphere or a CO_2_ atmosphere using 0.1 M tetrabutylammonium hexafluorophosphate (TBAPF_6_) as supporting electrolyte and it shows a similar electrochemical behaviour to the non-sulphur containing cyclam variant.^31,35^ (Fig. 2 and S1) In pure MeCN one reductive wave at −0.55 V *vs.* Fc/Fc^+^ is observed which can be attributed to the Ag(i) to Ag(0) reduction. This reduction is not completely reversible, and the oxidative wave at −0.33 V vanishes with higher scan rates (Fig. S2). At around 0 V *vs.* Fc/Fc^+^ a strong stripping signal is observed which can be attributed to the previous formation of a Ag(0) deposit on the surface of the working electrode which is reoxidized to Ag(i) and Ag(ii). The irreversible oxidation observed at +0.42 V likely corresponds to the Ag(i) to Ag(ii) transition, whereas the metal ion is likely to leave the coordination environment as a rather soft coordination environment of dithiacyclam is not suitable anymore for a hard cation. Purging the complex solution in pure MeCN with CO_2_ does not alter the CV. The addition of H_2_O in an Ar atmosphere shifts the Ag(i) reduction to a potential of −0.48 V. Additionally a beginning catalytic current, attributed to proton reduction, is observed at a potential of −2.11 V *vs.* Fc/Fc^+^. In the presence of CO_2_, a new reductive wave, induced by interaction with CO_2_, evolves at −1.75 V *vs.* Fc/Fc^+^ followed by a catalytic current. Additionally, a newly formed oxidative wave at +0.25 V *vs.* Fc/Fc^+^ can likely be attributed to the oxidation of a silver-(bi)carbonate species, as this signal is only observable in the presence of protons and CO_2_, as also observed for other silver complexes.^23^

The ability of Ag(dithiacyclam) to reduce CO_2_ in solution was investigated *via* controlled potential coulometry (CPC) at the potential of the second reductive wave in the presence of water and CO_2_, −1.85 V *vs.* Fc/Fc^+^. (Fig. S3) Catalysis was then performed for a duration of 24 h. The gaseous headspace was analysed two-hourly for the first 8 h and after 24 h *via* gas chromatography (GC). Only negligible quantities of CO with a FE_CO_ of 2% were observed after 24 h with large variations and poor reproducibility. The drop in total FE after 8 h to around 70% can be explained by the formation of (bi)carbonates, as is known for silver in acetonitrile, as well as electrolyte and catalyst degradation (*vide infra*).^36,37^ After electrolysis, a deposit was visible on the surface of the working electrode, which was verified to be elemental silver *via* scanning electron microscopy coupled with energy dispersive X-ray spectroscopy (SEM/EDX) (Fig. S4). As CO is solely evolving after more than 8 h of electrolysis, the formation of CO is most likely generated by this silver deposit, as also verified by a performed rinse test (Fig. S5). An electrode deposit was already observed after 2 h of electrolysis, as checked *via* SEM/EDX. This species is likely deposited Ag(dithiacyclam) as the EDX analysis revealed a clear overlap of silver, nitrogen and sulphur (Fig. S6), whereas the ligand breaks down in the ongoing electrolysis, indicating that the observed CO formation can be attributed to the elemental silver deposit formed later on. Thus, the rapid deposition accompanied by decomposition of the silver complex might have caused the big variations in FE as similar behaviour was previously observed for other molecular catalysts in solution.^38^

### Heterogeneous electrochemistry

Traditionally, the lack of activity of Ag(dithiacyclam) in homogeneous catalysis would discourage further investigation. This paradigm stems from the common practice of screening molecular complexes in homogeneous systems before translating them to heterogeneous eCO_2_R, thereby overlooking candidates that may only become active upon immobilization.^6,39^ Nevertheless, we pursued further studies on heterogeneous catalysis in ZGEs as we think Ag(dithiacyclam) is a promising complex for implementation into ZGEs. This assumption is supported by other molecular silver complexes that show high activity in ZGEs despite being only weakly active in solution, suggesting that Ag(dithiacyclam) may exhibit a similar behaviour.^10,22,24^

#### Electrolysis at low current densities (<100 mA cm^−2^)

First electrolysis experiments were performed in a ZGE equipped with a PiperION AEM (40 µm, Versogen, conditioned in 1 M KOH), a Ni-foam anode and 1 M KOH cycled as an anolyte (20 mL min^−1^). The cathode was fed with humidified CO_2_ (20 mL min^−1^, 100% rel. humidity) and the outlet gas stream was directly connected to a gas chromatograph (GC). Subsequent to a short electrochemical conditioning of the electrodes (details in the SI), electrolysis at 50 mA cm^−2^ was conducted for 1 h. The product gas composition was analysed every 30 min of electrolysis *via* inline GC. GDEs have been prepared by drop casting a catalytic ink onto a carbon cloth; the detailed procedure is described in the SI. In a first trial a Ag(dithiacyclam) loading of 0.5 mg cm^−2^ accompanied by 1.0 mg cm^−2^ carbon black (ENSACO 250G) as a carbon support and a 0.2 mg cm^−2^ SustainION XA-9 binder were chosen as GDE composition based on previous reports (Fig. S7).^24^ In strong contrast to the almost absent activity of the catalyst in solution, a high activity in eCO_2_R was observed reaching a remarkable FE_CO_ of 88% at 50 mA cm^−2^. *Via* changing the catalyst environment from an organic solvent to a solid, hydrophobic carbon matrix, the eCO_2_R behaviour of Ag(dithiacyclam) is completely changed. This activity increase can be attributed to beneficial properties of embedding molecular catalysts into GDEs: a very high local CO_2_ concentration, improved electron transfer by the carbon matrix and suppression of the competing HER by the alkaline and rather hydrophobic environment.^40–42^ Such a discrepancy in catalytic eCO_2_R activity between homogeneous electrolysis and zero-gap catalysis is rarely observed, since most molecular catalysts reported to be active in zero-gap electrolyzers, such as CoTPP or Ag(BIAN)s, also display at least moderate activity in homogeneous electrolysis.^22–24,43^

Since we want to exploit the full potential of Ag(dithiacyclam) as an eCO_2_R catalyst in GDEs, we wanted to determine the ideal carbon support as well as the best catalyst : carbon ratio since no clear trend exists in the literature – another issue of the minimal amount of holistic reports.^6^ Hence, we tested two different carbon black variants (ENSACO 250G and SuperP) which alter in key characteristics such as porosity, electrical conductivity and dispersibility in the solvent of catalytic inks.^10,44–46^ Besides carbon black we investigated multiwalled carbon nanotubes (MWCNTs) as they also exhibit a good conductivity.^6,22^ Under laboratory scale conditions as applied here, all investigated carbon supports and ratios thereof exhibit equally good performance reaching a FE_CO_ around 90% (Fig. S7). Nevertheless, a trend towards higher FE_CO_ is observed when SuperP with a loading of 0.5 mg cm^−2^ is applied as a carbon support reaching a maximal value of 97% which can be attributed to the high conductivity and large pore size.^44,45^ Thus, SuperP was chosen for the following studies. At lower current densities, the intrinsic activity of Ag(dithiacyclam) is sufficiently high that parameter-specific effects become difficult to disentangle, limiting the ability to draw meaningful conclusions. Therefore, the influence of varying GDE and process parameters was evaluated at higher current densities.

#### Minimizing the catalyst loading (≥100 mA cm^−2^)

After proving to be a suitable catalyst for eCO_2_R in ZGEs at low current densities (≤100 mA cm^−2^) further experiments targeting more industrially relevant current densities up to 500 mA cm^−2^ were performed.^1^ To reduce the chance of carbonate formation on the cathode side, which can block the pores of GDEs, the anolyte was changed to 1 M CsOH cycled at 20 mL min^−1^.^47,48^ Additionally, the anode was exchanged with IrO_2_ coated Ti-felt (1 mg cm^−2^) due to its high activity as an oxygen evolution catalyst.^49,50^ Molecular catalysts offer one big advantage compared to pure metal particles: they reach equal or even higher FE_CO_ values while bearing an overall lower metal content in the CL and thus reduce the need for precious metals.^24^ At room temperature three different catalyst loadings (0.05 mg cm^−2^, 0.125 mg cm^−2^ and 0.25 mg cm^−2^) have been tested while keeping the mass of SuperP carbon black (0.5 mg cm^−2^) and binder (SustainION XA-9, 0.4 mg) constant (Fig. S8). After the electrochemical conditioning of the electrodes consecutive electrolysis was performed at 100 mA cm^−2^, 300 mA cm^−2^ and 500 mA cm^−2^ for 30 min each following a product gas analysis *via* inline GC. At 100 mA cm^−2^ no significant difference in FE_CO_ is observable and selectivity close to 100% CO is generated for all loadings. Nevertheless, the performance rapidly decreased with low catalyst loadings as the current density was increased. The GDE with a loading of 0.25 mg cm^−2^ achieved the highest FE_CO_ of 28% at 500 mA cm^−2^ and the GDE with 0.125 mg cm^−2^ reached the lowest FE_CO_ of 9%. Yet, the measured cell voltage also increases with a higher catalyst loading, reaching −5.25 V. As a control experiment, GDEs with Ag NPs (<40 nm) with an equal metal content have been prepared and tested under identical conditions leading to smaller FE_CO_ at elevated current densities of 300 and 500 mA cm^−2^ (Fig. S9) yet maintaining a lower cell voltage than Ag(dithiacyclam). Since the ligand scaffold ensures a heteroatom rich environment, which herein benefits the CO generation activity, further testing towards even more industrially relevant conditions is pursued to assess transferability, identify potential mass-transport limitations, and evaluate catalyst stability.

#### Process optimization for electrolysis at 60 °C

**Fig. 3:**
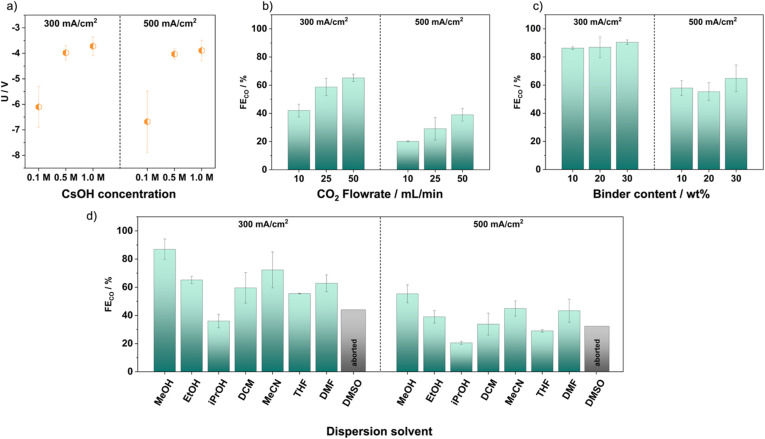
Selection of outstanding measurement data from electrolysis optimization processes at 60 °C at current densities of 300 mA cm^−2^ and 500 mA cm^−2^ achieved with a GDE comprising a Ag(dithiacyclam) loading of 0.5 mg cm^−2^. (a) Averaged cell voltages U achieved while applying different CsOH concentrations; FE_CO_ achieved while (b) applying the given *λ*_CO_2__ values; (c) comprising the given binder content in the CL; (d) utilizing the given solvent for dispersion of the catalytic ink.

As industrial scale electrolysis is beneficially run at elevated temperature, we performed the following catalysis experiments at 60 °C and high current densities of 300 mA cm^−2^ and 500 mA cm^−2^.^1^ These experiments were performed with a catalyst loading of 0.5 mg cm^−2^.The SuperP and SustainION XA-9 loadings of 0.5 mg cm^−2^ and 0.2 mg cm^−2^, respectively, were maintained for process optimization studies. A humidified (80% rel. humidity) CO_2_ flow of 50 mL min^−1^ was fed to the cathode as the starting point. At first the influence of cation crossover through the membrane from the anolyte was analysed by testing CsOH at concentrations of 0.1 M, 0.5 M and 1 M (Fig. S10). In terms of FE_CO_ all anolytes achieved values of 60% ± 7%; however, a decrease in cell voltage is observed with low concentrations leading to averaged cell voltages of −6.0 V, −3.85 V and −3.75 V at 300 mA cm^−2^ respective to concentrations of 0.1 M, 0.5 M and 1.0 M caused by lower solution resistance and preferred conditions for the oxygen evolution reaction (Fig. 3a).^25,51,52^ At 500 mA cm^−2^ the differences are even more prominent reaching a cell voltage of −6.5 V for 0.1 M CsOH and −3.9 V for 1.0 M CsOH (Fig. S10).

To ensure efficient proton supply, ionic conductivity and stabilization of the gas–liquid–solid interface required for efficient eCO_2_R, the next step of process optimization focused on the water delivery towards the cathode. Thus, different humidification percentages (0%, 25%, 50%, 80% and 100% rel. humidity) have been investigated by controlled variation of the bubbler temperature (Fig. S11). Below a humidification degree of 80% a FE_CO_ of 50% at 300 mA cm^−2^ is not exceeded indicating an insufficient water delivery. At 80% rel. humidity a maximum FE_CO_ of 65% is achieved. With an even higher humidification of 100% the FE_CO_ drops to 50% accompanied by a high cell voltage of −8.5 V most likely caused by electrode flooding due to large water amounts.

Aiming towards industrially relevant conditions, efficient CO_2_ utilization is desired. Thus the *λ*_CO_2__ value – the ratio between supplied and actually converted CO_2_ – should be as low as possible, yet it remains an uncommon metric in studies of molecular catalysts.^53,54^ Therefore, we decreased the CO_2_ feed to the cathode from 50 mL min^−1^ to 25 mL min^−1^ and 10 mL min^−1^ (Fig. 3b and S12). The corresponding *λ*_CO_2__ values at the applied current densities can be found in Table S1. With decreasing *λ*_CO_2__ values the selectivity for CO generation decreases, reaching only 20% and 29% FE_CO_ as observed for other molecular silver complexes in ZGEs.^24^ When the cathode feed stream is set to 10 mL min^−1^, the CO_2_ delivery to the cathode is insufficient as indicated by a FE_total_ of solely 73%. Even though high *λ*_CO_2__ values of 12 and 7 are needed to achieve good FE_CO_ values of 65% and 39%, respectively, it needs to be considered that the system is operating with a low silver loading of 0.22 mg cm^−2^ which is much lower than the commonly reported values of several milligrams per square centimetre of silver nanoparticle (NP) based GDEs.^54–57^ This low loading likely requires high CO_2_ feed rates to ensure that the generally low number of active sites is constantly supplied with CO_2_, to avoid local CO_2_ depletion and concentration gradients at the catalyst surface.

#### Optimization of dispersion solvent

After determining the optimal process parameters for catalysis at 60 °C, the GDE was optimized *via* ink engineering to control the electrode structure for optimal ion and electron transport and overall performance. Thus, the dispersion solvent and binder content have been varied and correlated with microstructure analysis of the GDEs. For all samples, 5 mg Ag(dithiacyclam) and 5 mg SuperP were dissolved in 2 mL solvent as before. Besides the primarily utilized EtOH, seven solvents of varying polarity were investigated (MeOH, iPrOH, MeCN, THF, DMF, DCM, and DMSO) while keeping the binder content constant at 20 wt% (Fig. 3d and S13). These solvents were chosen based on previous reports of dispersed molecular catalysts as well as good solubility (DCM, DMSO, and MeCN) of Ag(dithiacyclam).^10,43,58^ When DMSO was used, the measurements were aborted due to cell voltages exceeding 10 V during the first 20 min of electrolysis (Fig. S13h). While disassembling the cell it was observed that the GDE surface was “glued” to the membrane, potentially caused by interaction of leftover DMSO with the AEM, as the electrodes were only dried at 95 °C to maintain the integrity of SustainION XA-9 (Fig. S14). Thus, DMSO was thus excluded as suitable solvent for scale up processes.

**Fig. 4:**
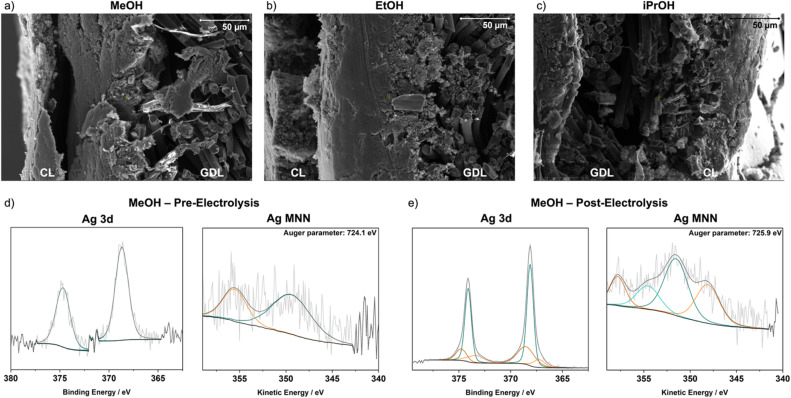
Recorded cross sectional SEM images at a magnification of 800× of pristine Ag(dithiacyclam) GDEs prepared with (a) MeOH; (b) EtOH; (c) iPrOH. Recorded Ag 3d and Ag NMM XPS spectra of (d) a pristine GDE prepared with MeOH and (e) a utilized GDE prepared with MeOH applied in short term catalysis at 300 mA cm^−2^ and 500 mA cm^−2^.

At 300 mA cm^−2^ the lowest FE_CO_ was achieved utilizing iPrOH with only 32%. As the observed variations in FE_CO_ are likely attributed to structural changes in the catalyst layer of the GDE, we performed a combination of SEM/EDX and computer tomography (CT) for microstructure analysis. As observed in both methods the CL of the iPrOH GDE is rather loosely packed (Fig. 4c) and comprises several big particle agglomerates (Fig. S15 and 16). In particular, the latter can explain the comparably bad performance in eCO_2_R as not many catalytic centres are accessible for CO_2_ (Fig. S16). All other tested solvents, except MeOH, range between FE_CO_ values of 56% and 72%. Of those mediocre performing GDEs, we decided to analyse the EtOH-based one *via* SEM/EDX and CT, as a FE_CO_ trend (higher FE_CO_ with higher polarity) is observed within the protic solvents. The CL of the EtOH-based GDE is packed a little denser (Fig. 4b), which can be beneficial for the conductivity (Fig. S17–S19). In the as-prepared state, fewer particle agglomerates are observed compared to the GDE prepared with iPrOH (Fig. S19). However, post-mortem analysis reveals that a larger number of particle agglomerates form in the EtOH based GDE during electrolysis (Fig. S20 and S21). From all studied dispersion solvents MeOH achieved the highest FE_CO_ of 87% at 300 mA cm^−2^ and even at a current density of 500 mA cm^−2^ the eCO_2_R dominates reaching a FE_CO_ of 55%. These findings are in line with previous reports, which determined MeOH as the most suitable dispersion solvent for a polar silver complex.^10^ The transferability of dispersion properties from one complex towards another simply based on the complex's polarity is a crucial finding to efficiently fine tune GDE fabrication. SEM/EDX and CT analysis of a MeOH-based GDE revealed that this high activity is likely caused by a very thin (Fig. 4a) and densely packed CL without visible agglomerates (Fig. S22–S24). Thus, a good CO_2_ distribution and conductivity are ensured. After electrolysis still no silver particles were observed maintaining an even CL surface (Fig. S25 and S26). However, it needs to be noted that for the latter a big part of the CL was removed, most likely during sample preparation, as this is also clearly observed in the CT images.

Besides the structural electrode features, the complex's integrity is of equal if not even greater interest. To analyse this, we decided on XPS spectroscopy, as we can determine the metal's oxidation state and coordination environment (Fig. 4d, e and S27–S30). No matter if the GDE was prepared with MeOH or EtOH the pristine GDEs show Ag 3d_5/2_ signals at 368.7/368.8 eV with Auger parameters of 724.1 and 723.8 eV, which is in line with XPS spectra reported for Ag(i) complexes on GDEs before.^10,24^ In addition the spectrum of the S 2p orbital shows signals for C–S–C as well as signals that can be attributed to a S–Ag bond.^59^ Post-electrolysis GDEs revealed the decomposition of the initial complex for both preparation methods, as three silver species are observed in the Ag 3d_5/2_ orbital spectra which can be attributed to a mixture of Ag(dithiacyclam), AgO and elemental silver (Fig. S29 and S30).^60,61^

In contrast to the degradation process in homogeneous electrolysis, leading to an elemental silver surface, the *in situ* generated silver particles within the GDE are supported by a heteroatom rich carbon matrix, which can enhance eCO_2_R activity.^62,63^ Furthermore, we would like to note that the observed silver species are not necessarily the species catalysing the eCO_2_R, as they potentially might be reoxidizing after the electrolysis is stopped, as also observed for other molecular catalysts.^64,65^ Even though a transformation of Ag(dithiacyclam) is observed, the silver species formed exhibits an elevated point towards eCO_2_R. Thus, the application of silver coordination compounds as precatalysts for eCO_2_R in ZGEs could become a promising approach to lower the silver content in GDEs. As we have recently highlighted, the final species – whether molecular or pre-catalyst – has little importance on the industrial stage, as long as the necessary target values are achieved.^6,10^

#### Optimization of binder content

Lastly, the binder content, previously equal to 20 wt%, was decreased- and increased to 10 wt% and 30 wt% (Fig. 3c and S31). With a binder content of 30 wt%, a slight increase in the FE_CO_ to 90% at 300 mA cm^−2^ was observed. However at 500 mA cm^−2^ a noteworthy increase to 65% was achieved. This improvement is likely attributed to a very dense yet highly porous surface structure combining excellent CO_2_ transport with high conductivity (Fig. S32). In contrast, no significant changes in FE_CO_ as well as electrode structure were observed with the lower binder content of 10 wt% (Fig. S33). Therefore, all subsequent experiments were conducted using the 30 wt% binder, as this composition proved to be the most effective. Under the herein optimized conditions, GDEs prepared with silver NPs and the carbon additive maintaining an equal silver loading to that of Ag(dithiacyclam) did not reach the FE_CO_ achieved by the molecular system reaching only FE_CO_ values of 26% and 9% at 300 mA cm^−2^ and 500 mA cm^−2^, respectively (Fig. S34). Overall, this stepwise optimization of electrolysis parameters and the GDE composition establishes Ag(dithiacyclam) as one of the most efficient molecular systems reported in ZGEs, while also enabling performance comparable to that of heterogeneous silver-based materials at high current densities (Table S2).^22,24,66–72^

#### Long-term catalysis

Currently there is still a big gap in research how molecular catalysts perform over several hours in ZGEs at industrially relevant current densities, as most studies have been performed at current densities ≤200 mA cm^−2^ and room temperature.^6^

**Fig. 5:**
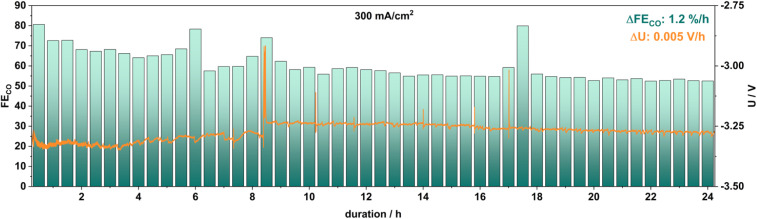
Measured FE_CO_ (green bars) and cell voltage U (orange line) over an electrolysis duration of 24 h applying an optimized GDE with a Ag(dithiacyclam) loading of 0.5 mg cm^−2^ and a binder loading of 30 wt% which was prepared using MeOH as dispersion solvent. The decay rates of FE_CO_ and U are given in the graph.

Hence, we tested the optimized GDEs over a time span of 24 h at 300 mA cm^−2^ (Fig. 5 and S35). Even after 24 h of constant electrolysis the FE_CO_ was still above 50%. Within the first 8 h of electrolysis the cell voltage fluctuates between −3.35 V and −3.3 V, following a quick jump towards −3.25 V. Since this jump is accompanied by a total FE exceeding 100% by far it can be assumed that this was caused by a pressure drop *i.e.* caused by temporary blocking of tubes by water droplets or carbonate salts. A similar jump is again observed after 16 h. Nevertheless, the cell voltage only slowly decreases from −3.23 V (8 h) to −3.27 V after 24 h, corresponding to a cell voltage decay rate of −0.005 V h^−1^.

Surface analysis of the GDE applied in 24 h electrolysis *via* SEM/EDX showed small, isolated silver agglomerates in the EDX elemental mapping without any traces of ligand (Fig. S36). CT analysis revealed that the GDL was blocked by particles, probably carbonate salts and CsOH (Fig. S37). This GDE flooding might have caused the drop in FE_CO_ as CO_2_ cannot be delivered sufficiently to the CL. Future efforts to improve long-term stability can focus on the reduction of salt formation, *i.e. via* utilization of a bipolar membrane in reverse-bias configuration or reduction of the cation concentration within the anolyte.^73^ Similar to the GDEs utilized in short-term testing, XPS analysis of the Ag 3d orbital revealed a mixture of Ag(dithiacyclam), AgO and elemental silver after 24 h of electrolysis (Fig. S38). Noteworthily, the formed mixture of silver compounds exhibits a higher activity after 24 h than a pristine electrode prepared with Ag NPs and carbon at the same metal loading, which solely reaches an FE_CO_ of 26% after 30 min of electrolysis (Fig. S34).

Overall, these promising values show how the direct incorporation of Ag(dithiacyclam) into GDEs gives another example of how efficiently transition metal complexes can perform in ZGEs under industrially relevant conditions, urging the community to further understand its importance as the first step to testing molecular electrocatalysts. Furthermore, we have shown that a usually overlooked component, the dispersion solvent, can drastically tune a catalyst performance as FE_CO_ values between 32% and 87% have been observed *via* changing the dispersion solvent as it influences the microstructure of the CL drastically. One final crucial milestone to achieve is long-term stability, which can be further improved by profoundly tailoring the binder type and alkali-ion concentration at the GDE interface, a topic that has only been investigated for metal nano-particles.^41,74–76^

## Conclusions

Herein we have shown a surprising trend that a transition metal complex, which does not homogeneously catalyse eCO_2_R, can nevertheless be a very potent catalyst in heterogeneous eCO_2_R. In essence the results redefine how we should test and optimize molecular electrocatalysts for potential large scale applications. Direct laboratory scale testing in a ZGE showed that Ag(dithiacyclam) can convert CO_2_ to CO efficiently with a FE_CO_ of 97% at a current density of 50 mA cm^−2^. In the first electrolysis experiments applying industrially relevant conditions (60 °C, ≥300 mA cm^−2^) a FE_CO_ of 65% was achieved at 300 mA cm^−2^. Notably, to maximise the activity of Ag(dithiacyclam) the catalytic ink composition was varied, as this is a crucial part of GDE fabrication which is often overlooked in the molecular community. In particular, the choice of dispersion solvent impacts the eCO_2_R performance of a GDE drastically resulting in a broad range of FE_CO_ covering values between 32% and 87% for eight different solvents. Of these, MeOH reached the highest FE_CO_ of 87% which was further improved to 90% *via* increasing the binder content.

With the optimized GDE composition, a remarkable FE_CO_ of 65% was achieved at 500 mA cm^−2^ in short term testing and a constant FE_CO_ above 50% was maintained for 24 h at 300 mA cm^−2^. *Via* SEM and CT analysis it was found that the dispersion solvent influences the structure of the CL and the formation of catalyst aggregates within it. XPS analysis revealed the partial transition of the molecular structure into a mixture of highly active silver particles which are more active than commercial silver NPs.

Hence, Ag(dithiacyclam) is a great example of the high potential of silver coordination compounds as catalyst precursors for eCO_2_R in ZGEs. On a further note our study points out important research questions which remain unanswered regarding the nature of the formed catalyst species and how they interact with the carbon support material. Moreover, this work stands to highlight how testing molecular electrocatalysts directly under heterogeneous conditions could be even more directed towards industrially relevant conditions, particularly, by placing the focus on catalytic ink optimization, as it drastically governs the CL structure and, as a result, the eCO_2_R performance as well.

## Author contributions

W. W.: conceptualization, formal analysis, investigation, methodology, validation, visualization, writing – original draft. K. P.: writing – review and editing. J. J.: supervision, writing – review and editing. U.-P. A.: conceptualization, supervision, funding acquisition, resources, writing – review and editing.

## Conflicts of interest

There are no conflicts to declare.

## Supplementary Material

SC-017-D6SC00957C-s001

## Data Availability

The data supporting this article have been included as part of the supplementary information (SI). Supplementary information: furthering electrochemical data, CT data, SEM/EDX data, XPS data, NMR spectra and further experimental details. See DOI: https://doi.org/10.1039/d6sc00957c.

## References

[cit1] Segets D., Andronescu C., Apfel U.-P. (2023). Nat. Commun..

[cit2] Stephens I. E. L., Chan K., Bagger A., Boettcher S. W., Bonin J., Boutin E., Buckley A. K., Buonsanti R., Cave E. R., Chang X., Chee S. W., Da Silva A. H. M., De Luna P., Einsle O., Endrődi B., Escudero-Escribano M., Ferreira De Araujo J. V., Figueiredo M. C., Hahn C., Hansen K. U., Haussener S., Hunegnaw S., Huo Z., Hwang Y. J., Janáky C., Jayathilake B. S., Jiao F., Jovanov Z. P., Karimi P., Koper M. T. M., Kuhl K. P., Lee W. H., Liang Z., Liu X., Ma S., Ma M., Oh H.-S., Robert M., Cuenya B. R., Rossmeisl J., Roy C., Ryan M. P., Sargent E. H., Sebastián-Pascual P., Seger B., Steier L., Strasser P., Varela A. S., Vos R. E., Wang X., Xu B., Yadegari H., Zhou Y. (2022). J. Phys. Energy.

[cit3] Tayyab M., Dreis M., Blaudszun D., Pellumbi K., Nzotcha U., Tempel H., Masood M. Q., Weinrich H., Stießel S., Junge Puring K., Eichel R.-A., Apfel U.-P. (2025). Energy Environ. Sci..

[cit4] Du S., Yang P., Li M., Tao L., Wang S., Liu Z.-Q. (2024). Chem. Commun..

[cit5] Küngas R. (2020). J. Electrochem. Soc..

[cit6] Wiesner W., Pellumbi K., Zimmermann I., Jökel J., Siegmund D., Apfel U.-P. (2025). Coord. Chem. Rev..

[cit7] Kibria M. G., Edwards J. P., Gabardo C. M., Dinh C., Seifitokaldani A., Sinton D., Sargent E. H. (2019). Adv. Mater..

[cit8] Siegmund D., Metz S., Peinecke V., Warner T. E., Cremers C., Grevé A., Smolinka T., Segets D., Apfel U.-P. (2021). JACS Au.

[cit9] Junge Puring K., Siegmund D., Timm J., Möllenbruck F., Schemme S., Marschall R., Apfel U. (2021). Adv. Sustain. Syst..

[cit10] Pellumbi K., Kräenbring M., Krisch D., Wiesner W., Sanden S., Siegmund D., Özcan F., Puring K. J., Cao R., Schöfberger W., Segets D., Apfel U. (2024). Small.

[cit11] Blommaert M. A., Sharifian R., Shah N. U., Nesbitt N. T., Smith W. A., Vermaas D. A. (2021). J. Mater. Chem. A.

[cit12] Francke R., Schille B., Roemelt M. (2018). Chem. Rev..

[cit13] Costentin C., Robert M., Savéant J.-M. (2015). Acc. Chem. Res..

[cit14] Gu S., Marianov A. N., Lu T., Zhong J. (2023). Chem. Eng. J..

[cit15] Boutin E., Merakeb L., Ma B., Boudy B., Wang M., Bonin J., Anxolabéhère-Mallart E., Robert M. (2020). Chem. Soc. Rev..

[cit16] Yin Z., Zhang M., Long Y., Lei H., Li X., Zhang X., Zhang W., Apfel U., Cao R. (2025). Angew. Chem., Int. Ed..

[cit17] Guo K., Li X., Lei H., Guo H., Jin X., Zhang X., Zhang W., Apfel U., Cao R. (2022). Angew. Chem..

[cit18] He H., Jian Y., Liu J., Yin Z., Lu Q., Lei H., Zhang W., Cao R. (2025). ChemSusChem.

[cit19] Han J., Wang N., Li X., Lei H., Wang Y., Guo H., Jin X., Zhang Q., Peng X., Zhang X.-P., Zhang W., Apfel U.-P., Cao R. (2022). eScience.

[cit20] Costentin C., Drouet S., Robert M., Savéant J.-M. (2012). Science.

[cit21] Azcarate I., Costentin C., Robert M., Savéant J.-M. (2016). J. Phys. Chem. C.

[cit22] Wiesner W., Arias J. Y. M., Jökel J., Cao R., Apfel U.-P. (2024). Chem. Commun..

[cit23] Krisch D., Sun H., Pellumbi K., Faust K., Apfel U.-P., Schöfberger W. (2022). Catalysts.

[cit24] Pellumbi K., Krisch D., Rettenmaier C., Awada H., Sun H., Song L., Sanden S. A., Hoof L., Messing L., Puring K. J., Siegmund D., Cuenya B. R., Schöfberger W., Apfel U.-P. (2023). Cell Rep. Phys. Sci..

[cit25] Siritanaratkul B., Khan M. D., Yu E. H., Cowan A. J. (2024). Philos. Trans. R. Soc., A.

[cit26] Biemolt J., Singh J., Prats Vergel G., Pelzer H. M., Burdyny T. (2025). ACS Energy Lett..

[cit27] Idros M. N., Wu Y., Duignan T., Li M., Cartmill H., Maglaya I., Burdyny T., Wang G., Rufford T. E. (2023). ACS Appl. Mater. Interfaces.

[cit28] Beley M., Collin J.-P., Ruppert R., Sauvage J.-P. (1984). J. Chem. Soc., Chem. Commun..

[cit29] Beley M., Collin J. P., Ruppert R., Sauvage J. P. (1986). J. Am. Chem. Soc..

[cit30] Siritanaratkul B., Forster M., Greenwell F., Sharma P. K., Yu E. H., Cowan A. J. (2022). J. Am. Chem. Soc..

[cit31] Ski D. S., Grzejdziak A. (2002). J. Inclusion Phenom. Macrocycl. Chem..

[cit32] Gerschel P., Warm K., Farquhar E. R., Englert U., Reback M. L., Siegmund D., Ray K., Apfel U.-P. (2019). Dalton Trans..

[cit33] Iffland L., Siegmund D., Apfel U. (2020). Z. Anorg. Allg. Chem..

[cit34] Johnson A., Iffland L., Singh K., Apfel U.-P., Suntharalingam K. (2021). Dalton Trans..

[cit35] Grzejdziak A. (1994). Monatsh. Chem..

[cit36] Figueiredo M. C., Ledezma-Yanez I., Koper M. T. M. (2016). ACS Catal..

[cit37] Mairegger T., Li H., Grießer C., Winkler D., Filser J., Hörmann N. G., Reuter K., Kunze-Liebhäuser J. (2023). ACS Catal..

[cit38] Guseva T., Bera A., Ludwig V., Siegmund D., Gerschel P., Ray K., Apfel U. (2025). Eur. J. Inorg. Chem..

[cit39] Grammatico D., Bagnall A. J., Riccardi L., Fontecave M., Su B., Billon L. (2022). Angew. Chem., Int. Ed..

[cit40] Marianov A. N., Jiang Y. (2022). Acc. Mater. Res..

[cit41] Rabiee H., Ge L., Zhang X., Hu S., Li M., Yuan Z. (2021). Energy Environ. Sci..

[cit42] Xing Z., Hu L., Ripatti D. S., Hu X., Feng X. (2021). Nat. Commun..

[cit43] Hu X., Rønne M. H., Pedersen S. U., Skrydstrup T., Daasbjerg K. (2017). Angew. Chem., Int. Ed..

[cit44] Wutthiprom J., Phattharasupakun N., Sawangphruk M. (2017). ACS Omega.

[cit45] Li O. L. and Ishizaki T., in Emerging Materials for Energy Conversion and Storage, Elsevier, 2018, pp. 115–152

[cit46] Amin A. S., Caidi A., Lange T., Radev I., Sandbeck D. J. S., Philippi W., Kräenbring M., Öztürk M., Peinecke V., Lerche D., Özcan F., Segets D. (2025). Part. Part. Syst. Charact..

[cit47] Moss A. B., Garg S., Mirolo M., Giron Rodriguez C. A., Ilvonen R., Chorkendorff I., Drnec J., Seger B. (2023). Joule.

[cit48] Cofell E. R., Nwabara U. O., Bhargava S. S., Henckel D. E., Kenis P. J. A. (2021). ACS Appl. Mater. Interfaces.

[cit49] Vass Á., Kormányos A., Kószó Z., Endrődi B., Janáky C. (2022). ACS Catal..

[cit50] Masel R. I., Liu Z., Yang H., Kaczur J. J., Carrillo D., Ren S., Salvatore D., Berlinguette C. P. (2021). Nat. Nanotechnol..

[cit51] Siritanaratkul B., Sharma P. K., Yu E. H., Cowan A. J. (2023). Adv. Mater. Interfaces.

[cit52] Yang K., Li M., Subramanian S., Blommaert M. A., Smith W. A., Burdyny T. (2021). ACS Energy Lett..

[cit53] Shin H., Hansen K. U., Jiao F. (2021). Nat. Sustain..

[cit54] Heuser S., Hoof L., Pellumbi K., Oberndorf J. N., Krämer L., Blaudszun D., Puring K. J., Prokein M., Mölders N., Kilzer A., Petermann M., Apfel U.-P. (2025). Chem Catal..

[cit55] Chanda V., Blaudszun D., Hoof L., Sanjuán I., Pellumbi K., Junge Puring K., Andronescu C., Apfel U. (2024). Chemelectrochem.

[cit56] Mowbray B. A. W., Dvorak D. J., Taherimakhsousi N., Berlinguette C. P. (2021). Energy Fuels.

[cit57] Samu A. A., Szenti I., Kukovecz Á., Endrődi B., Janáky C. (2023). Commun. Chem..

[cit58] Sun L., Reddu V., Xi S., Dai C., Sheng Y., Su T., Fisher A. C., Wang X. (2022). Adv. Energy Mater..

[cit59] Best S. A., Brant P., Feltham R. D., Rauchfuss T. B., Roundhill D. M., Walton R. A. (1977). Inorg. Chem..

[cit60] Kaushik V. K. (1991). J. Electron Spectrosc. Relat. Phenom..

[cit61] Ferraria A. M., Carapeto A. P., Botelho Do Rego A. M. (2012). Vacuum.

[cit62] Karapinar D., Huan N. T., Ranjbar Sahraie N., Li J., Wakerley D., Touati N., Zanna S., Taverna D., Galvão Tizei L. H., Zitolo A., Jaouen F., Mougel V., Fontecave M. (2019). Angew. Chem., Int. Ed..

[cit63] Liu L., Li M., Chen F., Huang H. (2023). Small Struct..

[cit64] Boutin E., Salamé A., Robert M. (2022). Nat. Commun..

[cit65] Weng Z., Wu Y., Wang M., Jiang J., Yang K., Huo S., Wang X.-F., Ma Q., Brudvig G. W., Batista V. S., Liang Y., Feng Z., Wang H. (2018). Nat. Commun..

[cit66] Ren S., Joulié D., Salvatore D., Torbensen K., Wang M., Robert M., Berlinguette C. P. (2019). Science.

[cit67] Wiesner W., Wilhelm C., Hoffmann R. C., Stahl P., Pellumbi K., Jökel J., Ivanović-Burmazović I., Apfel U.-P. (2026). Angew. Chem., Int. Ed..

[cit68] Yari F., Aljabour A., Awada H., Michalke J., Kumari N., Coskun-Aljabour H., Roy S., Krisch D., Schöfberger W. (2024). ACS Appl. Energy Mater..

[cit69] Wang R., Haspel H., Pustovarenko A., Dikhtiarenko A., Russkikh A., Shterk G., Osadchii D., Ould-Chikh S., Ma M., Smith W. A., Takanabe K., Kapteijn F., Gascon J. (2019). ACS Energy Lett..

[cit70] Seteiz K., Häberlein J. N., Heizmann P. A., Disch J., Vierrath S. (2023). RSC Adv..

[cit71] Lee W. H., Ko Y.-J., Choi Y., Lee S. Y., Choi C. H., Hwang Y. J., Min B. K., Strasser P., Oh H.-S. (2020). Nano Energy.

[cit72] Seteiz K., Grammel H., Häberlein J. N., Heizmann P. A., Metzler L., Rusitov D., Günthel M., Knäbbeler-Buß M., Vierrath S., Disch J. (2025). Nano Energy.

[cit73] Pärnamäe R., Mareev S., Nikonenko V., Melnikov S., Sheldeshov N., Zabolotskii V., Hamelers H. V. M., Tedesco M. (2021). J. Membr. Sci..

[cit74] Alinejad S., Quinson J., Li Y., Kong Y., Reichenberger S., Barcikowski S., Broekmann P., Arenz M. (2024). J. Catal..

[cit75] Hoof L., Pellumbi K., Güney D. C., Blaudszun D., Bommas F., Siegmund D., Puring K. J., Cao R., Weber K., Apfel U.-P. (2025). RSC Sustain..

[cit76] Disch J., Bohn L., Metzler L., Vierrath S. (2023). J. Mater. Chem. A.

