# Supplementary material for: From overlooked to outstanding: a molecular silver complex in heterogeneous CO2 electroreduction
Source: Chem Sci. 2026 Jun 4;17(29):14336–45. doi: 10.1039/d6sc00957c (PMC13271064; doi:10.1039/d6sc00957c)
Supplement: SC-017-D6SC00957C-s001 [file SC-017-D6SC00957C-s001.pdf]

Supplementary Information (SI) for Chemical Science.
This journal is © The Royal Society of Chemistry 2026

Supplementary Information

From Overlooked to Outstanding: A Molecular Silver Complex in Heterogeneous CO_2 Electroreduction

Wiebke Wiesner[a], Kevinjeorjios Pellumbi[b], Julia Jökel[b] and Ulf-Peter Apfel*[a,b]

[a] Ruhr-Universität Bochum, Fakultät für Chemie und Biochemie, Activation of Small Molecules/Technical Electrochemistry, Universitätsstr. 150, 44801 Bochum Germany
[b] Fraunhofer UMSICHT, Department Powert-to-Chemicals, Osterfelderstr. 3, 46047 Oberhausen Germany

Inhaltsverzeichnis

EXPERIMENTAL 1

SYNTHESIS 1
HOMOGENEOUS ELECTROCHEMISTRY 2
GAS CHROMATOGRAPHY FOR HOMOGENEOUS ELECTROLYSIS 2
PREPARATION OF GAS DIFFUSION ELECTRODES 2
PREPARATION OF IrO_2 COATED ANODES 3
HETEROGENEOUS ELECTROCHEMISTRY 3
ONLINE GAS CHROMATOGRAPHY 4
SCANNING ELECTRON MICROSCOPY 4
X-RAY PHOTOELECTRON SPECTROSCOPY 4
COMPUTER TOMOGRAPHY 5

ADDITIONAL FIGURES 5

ADDITIONAL TABLES 27

REFERENCES 28

Experimental

Synthesis

All used chemicals were bought from commercial vendors and used without further purification. Solvents were dried using standard procedures and stored under an Ar atmosphere. The **dithiacyclam** ligand (1,8-dithia-4,11-diazacyclotetradecane) and the **Ag(dithiacyclam)** complex were synthesized according to literature known procedures, and their successful formation was verified *via* NMR spectroscopy and ESI-MS.[1,2]

Homogeneous Electrochemistry

Homogeneous electrochemical measurements were performed using a *PalmSens3* or *PalmSens4* potentiostat and a common three electrode set up. A 3 mm diameter glassy carbon electrode was used as working electrode (WE), a silver wire as pseudo-reference electrode (RE) and a platinum wire as counter electrode (CE). The WE was prepared by polishing with sandpaper of different textures, following sonication in acetonitrile for 10 min. Tetrabutylammonium hexafluorophosphate ($TBAPF_6$) was used the supporting electrolyte with a concentration of 0.1M. All potentials were referenced against the ferrocene/ferrocenium couple (Fc/Fc+). Controlled potential coulometry (CPC) was performed applying a potential of -1.59 V for the 24 h, gaseous samples of the headspace were analyzed every 2 h for the first 8 h and again after 24 h *via* hand injection into a Shimadzu BID-2010 gas chromatograph.

Rinse tests were performed *via* performing linear sweep voltammetry (LSV) measurements of the WE previously applied in CPC after a defined amount of time to measure the current response of potentially formed electrode deposit.

Gas Chromatography for homogeneous electrolysis

Gas chromatography of the headspace was performed by hand injection on a Shimadzu GC-2010 Pro equipped with a Shimadzu BID-2010 Plus barrier discharge ionization detector (BID). The gas phase separation was performed using a SPL injector and a Carboxen 1010 PLOT fused silica capillary GC column (L x I.D. 30 m x 0.32 mm, average thickness 15 µm). Solvents were cut off previously by a SH-Rxi-1ms fused silica capillary GC column (L x I.D. 30 m x 0.32 mm, average thickness 1.0 µm). Helium was used as carrier gas. Detection of the following compounds was possible *via* GC-BID: H_2, O_2, CO, CH_4, C_2H_4 and C_2H_6.

Preparation of Gas Diffusion Electrodes

Standard procedure for experiments at r.t. A catalytic ink was prepared by sonicating the desired amount of catalyst and the desired amount of carbon support (either carbon black or MWCNTS, diameter of 10-20 nm purchased from TCI) and 50 µL binder solution (either SustainIon XA-9 Binder 5 wt% in EtOH, purchased from Dioxide Materials or Nafion 5 wt% in EtOH, purchased from Sigma Aldrich respectively) in 2 mL of EtOH for 30 min. Following the catalytic ink was drop casted onto circular carbon cloth (W1S1011) gas diffusion layers (diameter of 1.6 cm, purchased from Freudenberg) at a temperature of 90 °C. Drop casting was performed by dropping the ink in 100 µL steps evenly onto the carbon cloth until the desired loading of per electrode was reached which was controlled via weighing before and after drop casting.

Standard procedure for experiments at 60 °C and high current densities. A catalytic ink was prepared by sonicating 5 mg of Ag(dithiacyclam), 5 mg carbon black (SuperP) and 50 µL binder solution (SustainIon XA-9 Binder 5 wt% in EtOH, purchased from Dioxide Materials) in 2 mL of EtOH (if not stated otherwise within the main text) for 30 min. Afterwards the catalytic ink was drop casted onto circular carbon cloth (W1S1011) gas diffusion layers (diameter of 1.6 cm, purchased from Freudenberg) at a temperature of

90 °C. Drop casting was performed by dropping the ink in 100 µL steps evenly onto the carbon cloth until the desired loading of per electrode was reached which was controlled gravimetrically.

Preparation of IrO_2 coated Anodes

To prepare 20 circular Ti-felt anodes (thickness 1 mm, 1.6 cm diameter) a catalytic ink was prepared made of 90 mg IrO_2 which was dispersed in 1.5 mL HPLC grade water. Following 4.36 mL isopropanol and 17.5 µL Triton X-100 were added. The mixture was sonicated for 15 min. Afterwards 45 µL of a 30 wt% PTFE solution (Quintech) was added was constantly stirred. To prepare the anodes the catalytic ink was manually spray coated onto the Ti-felts with an Iwata Eclipse spray gun at a pressure of 1 bar. The Ti-felts were heated to 110 °C during the process. The loading (1 mg/cm^2) was controlled *via* weighing. After spray coating the Triton X-100 was burned out at 300 °C for 10 min.

Heterogeneous Electrochemistry

Electrocatalysis was performed in an in-house built zero-gap electrolyzer consisting of stainless-steel endplates, insulation plates made from PTFE (1 mm thickness), copper current collectors, titanium-based flow fields (linear for anode side, serpentine for cathode side) and PTFE gaskets to hold the electrodes in place, as reported elsewhere.[3] The cell was tightened with a torque to 5 Nm. During all measurements a PiperION anion exchange membrane (40 µm thickness, bought from Versogen, soaked in the used anolyte for 24 h prior to use) was used. All aqueous solutions were prepared with HPLC grade water. In all experiments with current densities ≤100 mA/cm^2 untreated Ni foam (thickness >2 mm, diameter 1.6 cm, purchased from Goodfellow) was used as anode. In all experiments with current densities ≥100 mA/cm^2 IrO_2 coated Ti-felt (1 mg/cm^2, diameter 1.6 cm) was used as anode. Anolytes were cycled using a peristaltic pump (GILSON Minipuls3) with a speed of 20 mL/min. The CO_2 gas stream was humidified *via* purging through heatable bubbler filled with HPLC grade water prior to the cell inlet. Experiment at 60 °C were conducted in an oven to heat cell and anolyte. A GAMRY Reference 1010B potentiostat was used during all electrochemical experiments.
Prior to electrocatalysis at r.t. the electrodes were conditioned by applying current densities of 10 mA/cm^2, 20 mA/cm^2, 30 mA/cm^2, 40 mA/cm^2 and 50 mA/cm^2 for one minute each. Afterwards chronopotentiometric measurements at the given current densities were applied consecutively for 60 min each. Each 30 min the outlet gas stream was analysed *via* Online gas chromatography.
Prior to electrocatalysis at 60 °C the electrodes were conditioned by applying current densities from 20 mA/cm^2 to 300 mA/cm^2 in steps of 20 mA/cm^2 for 30 s each. Afterwards a chronopotentiometric measurement at 300 mA/cm^2 was run for 30 min following product gas analysis *via* Online GC. Subsequently the applied current density was increased stepwise (20 mA/cm^2 steps) until a current density of 500 mA/cm^2 is reached, following 30 min electrolysis at 500 mA/cm^2 with a subsequent product gas analysis *via* Online GC

Online Gas Chromatography

For heterogeneous electrolysis experiments at r.t. the outlet gas stream of the ZGE was connected to an Agilent Technologies 7820A gas chromatograph equipped with two columns (HP-Plot Q and a HP-Molsieve 5 Å column for product separation) and a flame ionization detector (FID), a thermal conductivity detector (TCD) and a Ni-based methanizer. The carrier gas is Ar. The sample flow towards the column was regulated by a Bronkhorst EL-FLOW mass flow controller (1.67 mL/min). GC Samples of the cell outlet gas stream were analysed every 30 min and the resulting chromatograms were evaluated using the software UniChrom V. Detection of the following compounds was possible using the FID/TCD: H_2, N_2, CO, CH_4, C_2H_4 and C_2H_6.

Online gas chromatography for the electrocatalysis performed at 60°C was done using a Shimadzu Nexis GC-2030 which is equipped with two columns (a SH-I-1MS column (L x I.D. 30 m x 0.32 mm, average thickness 1 μm) and a Carboxen 1010 Plot column (L x I.D. 30 m x 0.32 mm, average thickness 15 μm)) followed by a particle trap in front of the Barrier Ion Discharge (BID) detector. The carrier gas is He. Detection of the following compounds was possible *via* GC-BID: H_2, Ar, CO, CH_4, C_2H_4 and C_2H_6.

The Faradaic Efficiency (FE) of gaseous products was calculated using the following equation:

$$FE(\%) = \frac{z \times n \times F}{Q} \times 100\% \quad (1)$$

Herein *z* is the number of transferred electrons during the process, *n* the amount of substance, *F* the faraday constant and *Q* the total charge passed.

To calculate the CO_2 excess λ_{CO2} the following equation was use the following equation

$$\lambda_{CO2} = \frac{n_{CO2}}{n_{CO_{max}}} = \frac{n_{CO2} \times z \times F}{I_{cell}} \quad (2)$$

Wherein n_{CO2} is the molar CO_2 flow rate (mol/s) and n_{COmax} is the maximum molar CO flow (mol/s) produced. Z is the number of electrons transferred - herein 2, F is the Faraday constant and I_{cell} is the applied current.

The relative humidity was calculated using the following equation:

$$rel. humidity = \frac{p_{vap}(bubbler)}{p_{vap}(cell)} \times 100\% \quad (3)$$

Here p_{vap}(bubbler) corresponds to the vapor pressure at the applied temperature of the bubbler and p_{vap}(cell) corresponds to the vapor pressure at the cell temperature.

Scanning electron microscopy

Scanning electron microscopy (SEM) was performed on ZEISS Gemini 2 Merlin HR-FESEM equipped with an OXFORD AZtecEnergy X-ray microanalysis system for energy dispersive X-ray spectroscopy (EDX) or a ThermoFischer Dualbeam FIB-SEM SCIO2 machine equipped with an Ultimax silicon drift detector (170 mm2, Oxford Instrument) for EDX measurements. SEM images were recorded with an acceleration voltage of 5 kV, EDX measurements with a voltage of 20 kV

X-ray photoelectron spectroscopy

The X-ray photoelectron spectra (XPS) of the electrodes used in this work were acquired using a Nexsa G2 Surface Analysis System (ThermoFischer) with monochromated and microfocused Al Kα-rays. The detector was 128-channel together with a 180°, double-

focusing, hemispherical analyser. All samples were analysed with a bandpass energy of 50.00 eV and the resulting peaks were fitted using the software CasaXPS 2.4.24. A Shirely-type background and a Lorentzian line shape were applied. Each spectrum was calibrated against adventitious carbon, which was set to 284.8 eV. Peaks that were not assigned as satellite peaks were limited to a full width at half maximum of 2.0 eV.

Computer tomography

X-Ray Computer Tomography (CT) was performed with a Bruker Skyscan 2214 CMOS edition scanner equipped with a Hamamatsu L10711 tungsten source. Electrode samples of roughly 2 mm width were cut and clamped in a custom sample holder for scanning. X-ray images were recorded at 55 kV and 90 µA with an image pixel size of 0.91 µm. A flat field correction was conducted before each scan including an automatic adjustment of the exposure time to values of 846 – 939 ms. Each scan acquired 1801 frames and averaged 4 of them for each position of a full rotation in 0.2° steps. Reconstruction and analysis were performed using Bruker software NRecon and CTVox.

Additional Figures

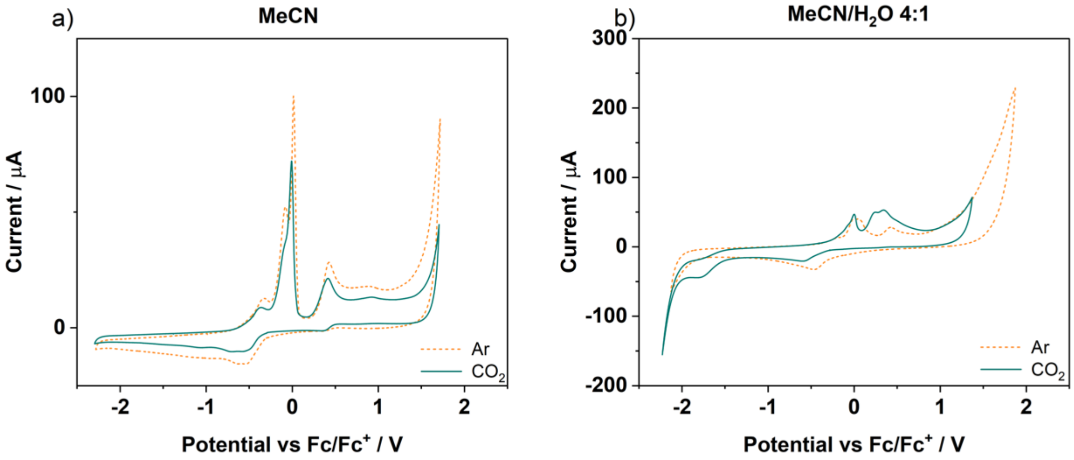

Figure S1. CVs of **Ag(dithiacyclam)** (0.1 mM in MeCN) recorded at a scan speed of 100 mV/s in A) MeCN and B) MeCN/H_2O 4:1 with 0.1 M $TBAPF_6$ as supporting electrolyte. Either in an Ar (orange) or CO_2 (dark green) atmosphere.

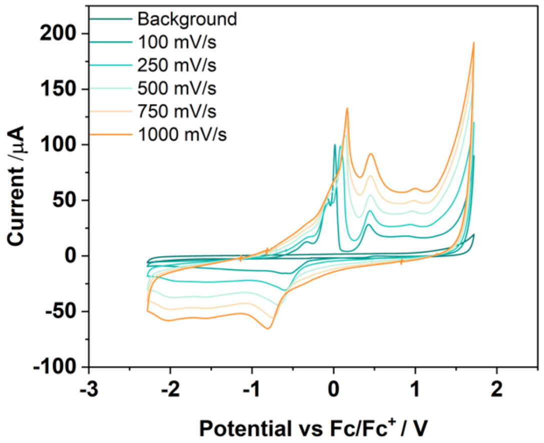

Figure S2. CVs of **Ag(dithiacyclam)** (0.1 mM in MeCN) recorded at the given scan speed in MeCN with 0.1 M TBAPF$_6$ as supporting electrolyte in an Ar atmosphere.

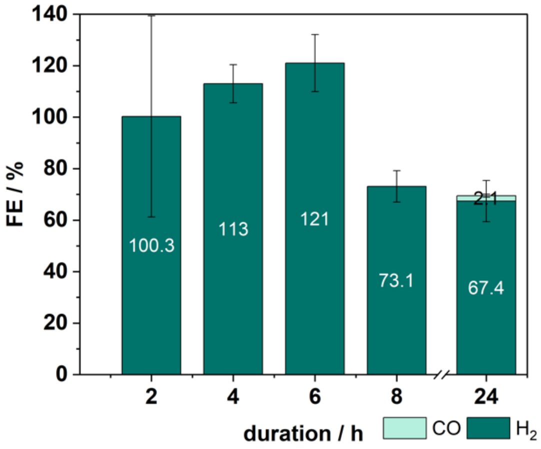

Figure S3. Bar chart of FE$_{H2}$ (dark green) and FE$_{CO}$ (light green) achieved by homogeneous electrolysis performed at a potential of -1.59 V in MeCN/H_2O with 0.1 M TBAPF$_6$ as supporting electrolyte.

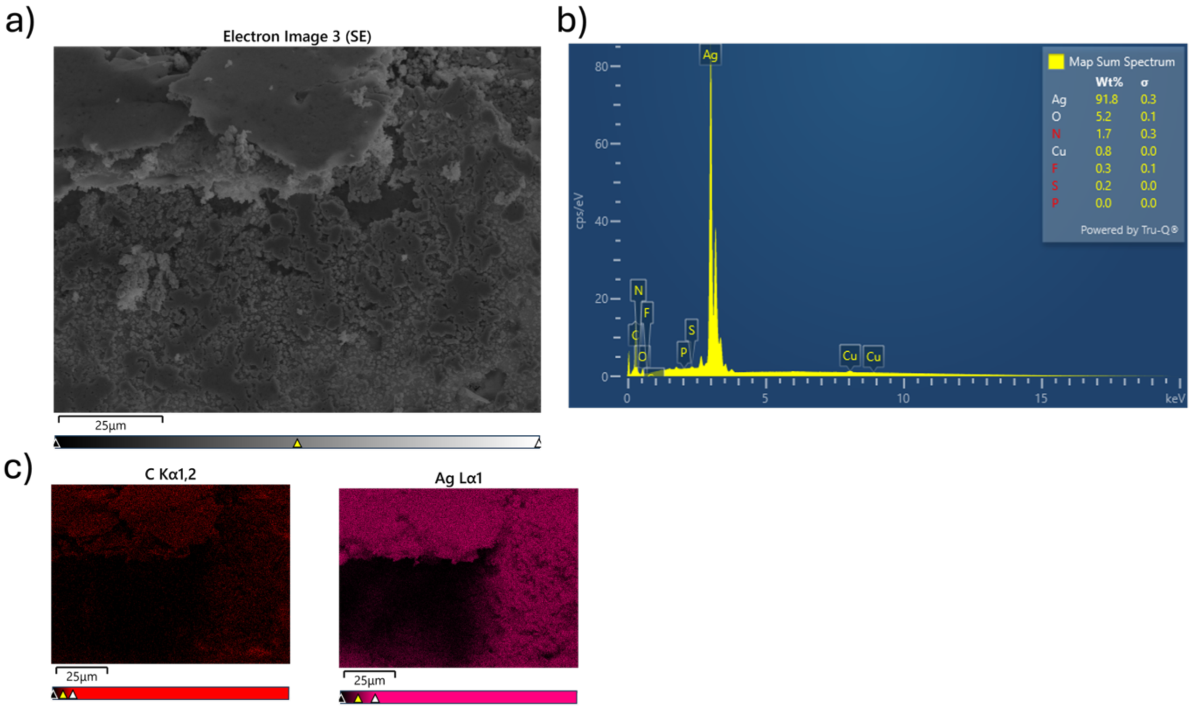

Figure S4. SEM/EDX images of the deposit formed on the WE surface after 24 h of homogeneous electrolysis performed at -1.59 V. A) SEM image at a magnification of ; B) EDX spectrum; C) elemental mapping.

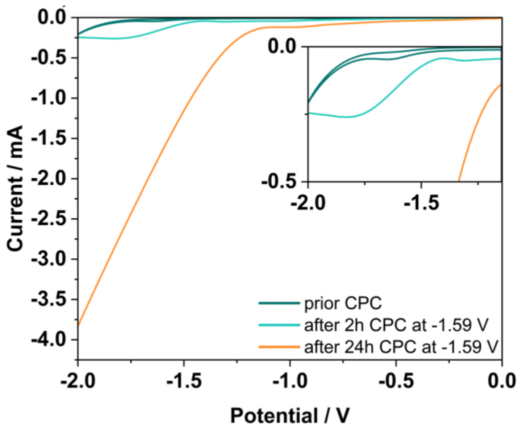

Figure S5. Results of the performed rinse test showing the comparison of a CV recorded of **Ag(dithiacyclam)** (dark green line) and LSVs recorded of the working after being applied in CPC measurements for 2 h (light green line) or 24 h (orange line) at an applied potential of -1.59 V in CO_2 purged MeCN/H_2O 4:1 with 0.1 M TBAPF6$_6$ as supporting electrolyte.

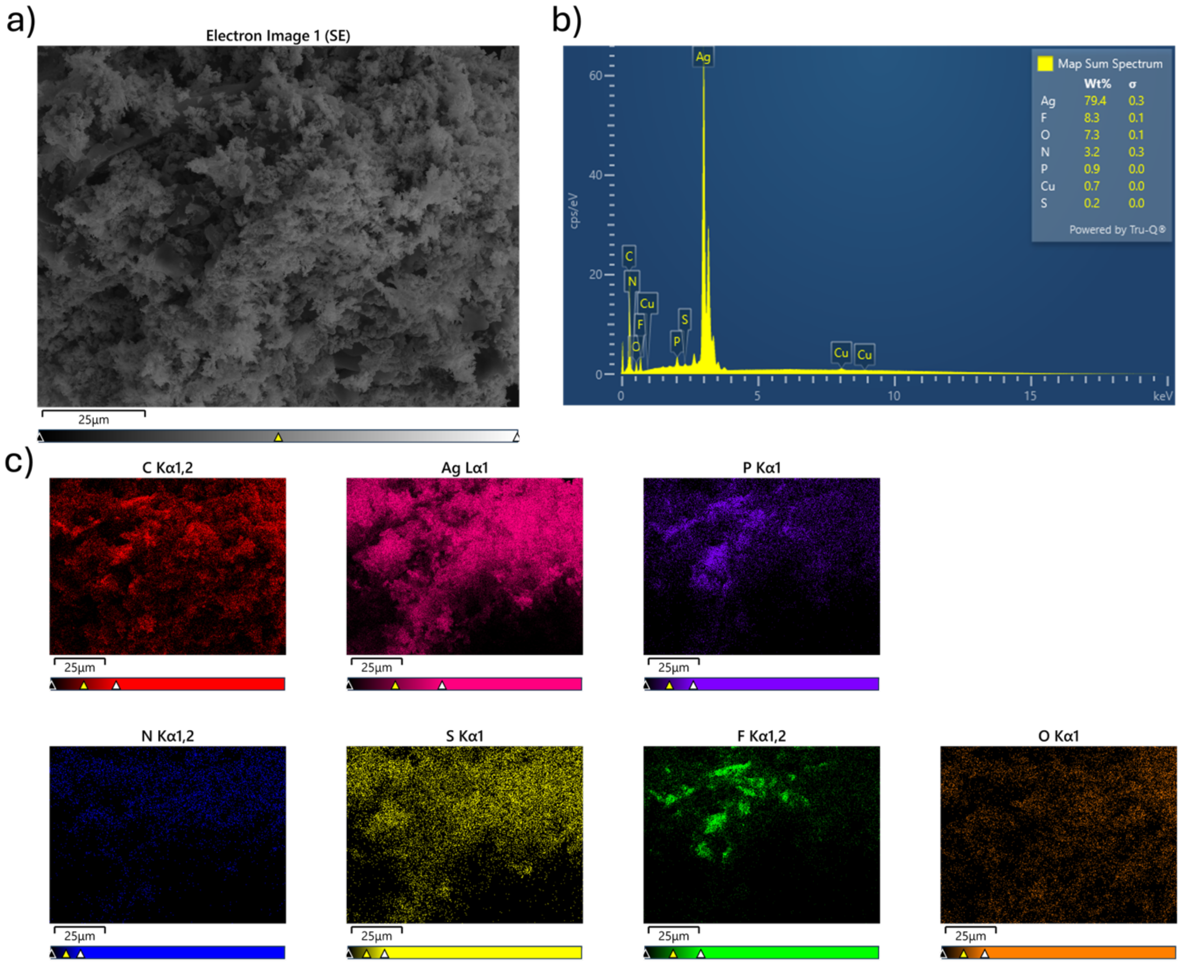

Figure S6. SEM/EDX images of the deposit formed on the WE surface after 2 h of homogeneous electrolysis performed at -1.59 V. A) SEM image at a magnification of ; B) EDX spectrum; C) elemental mapping.

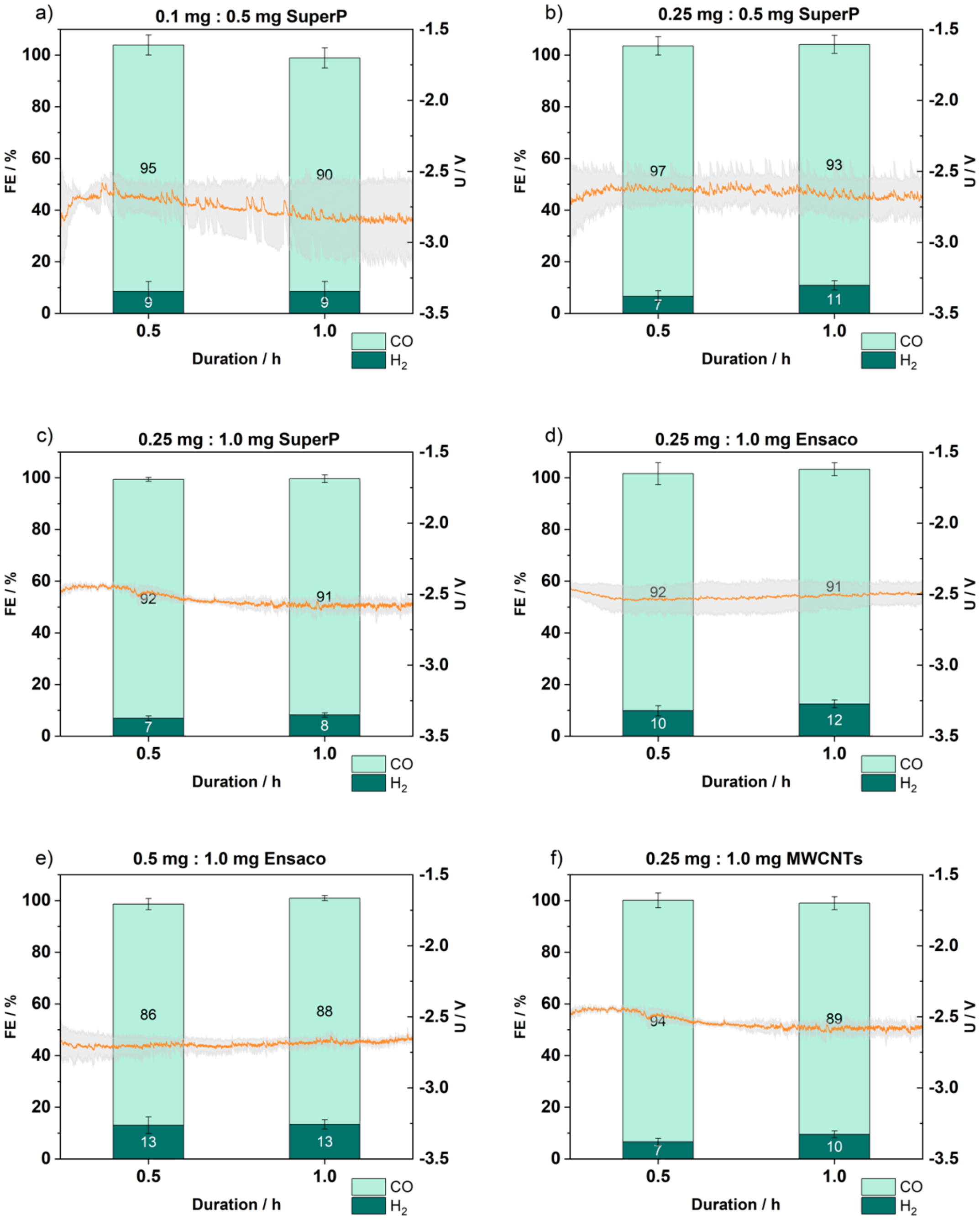

Figure S7. Detailed overview of the achieved FE_{CO} (dark green) and FE_{H2} (light green) pictured as bar chart with the corresponding cell voltage (orange line) achieved in r.t. catalysis at 50 mA/cm^2 for 1 h. The GDE loading /cm^2 is given as **Ag(dithiacyclam)**:Carbon Support within the graph.

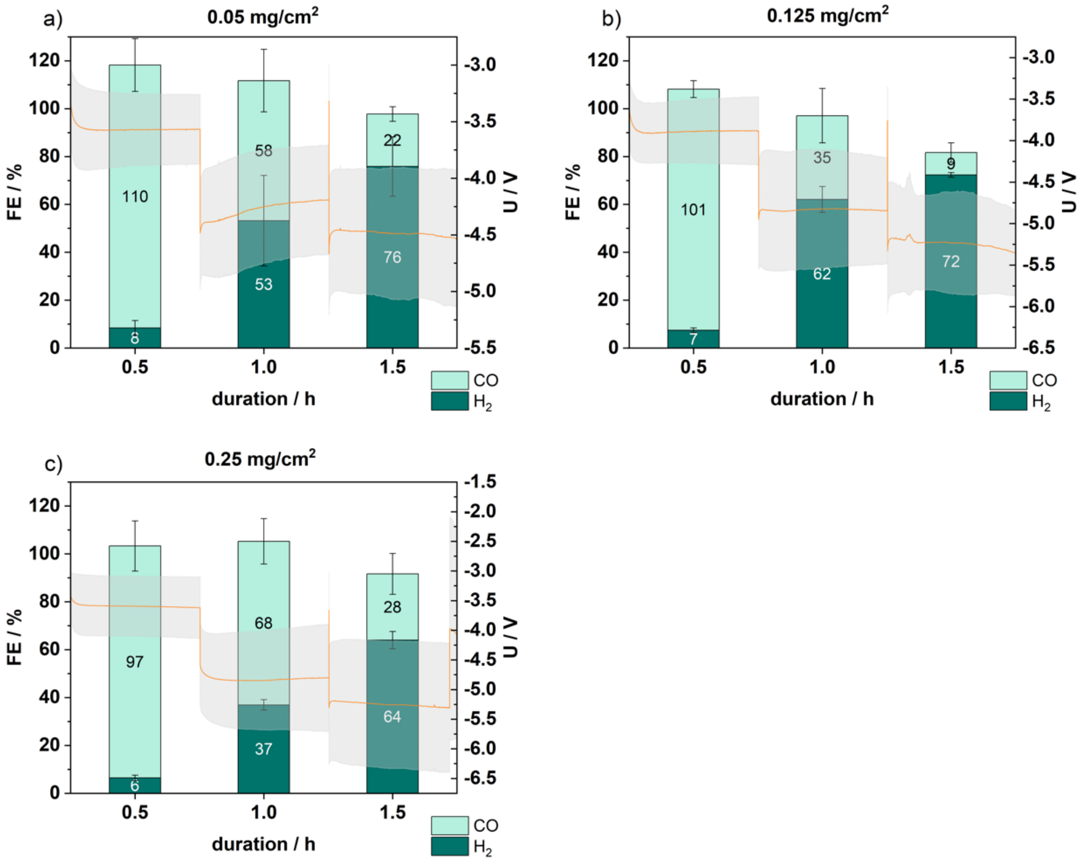

Figure S8. Detailed overview of the achieved FE_{CO} (dark green) and FE_{H2} (light green) pictured as bar chart with the corresponding cell voltage (orange line) achieved in r.t. catalysis at 100, 300 and 500 mA/cm² for 30 min each. The GDEs were coated with 0.5 mg/cm² of SuperP as carbon support and the given loading of **Ag(dithiacyclam)**. 1 M CsOH was used as anolyte.

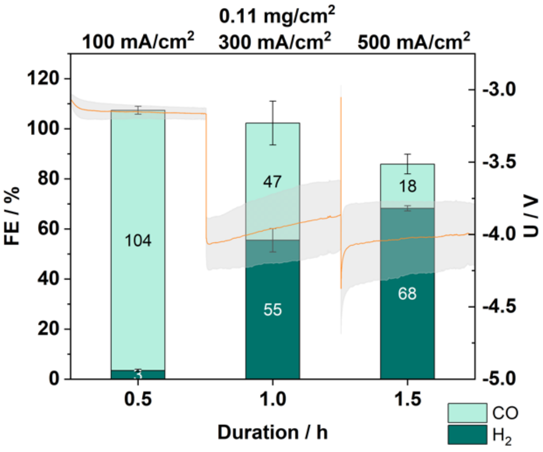

Figure S9. Detailed overview of the achieved FE_{CO} (dark green) and FE_{H2} (light green) pictured as bar chart with the corresponding cell voltage (orange line) achieved in r.t. catalysis at 100, 300 and 500 mA/cm² for 30 min each. The GDEs were coated with 0.5 mg/cm² of SuperP as carbon support and 0.11 mg/cm² Ag NPs (<40 nm). 1 M CsOH was used as anolyte.

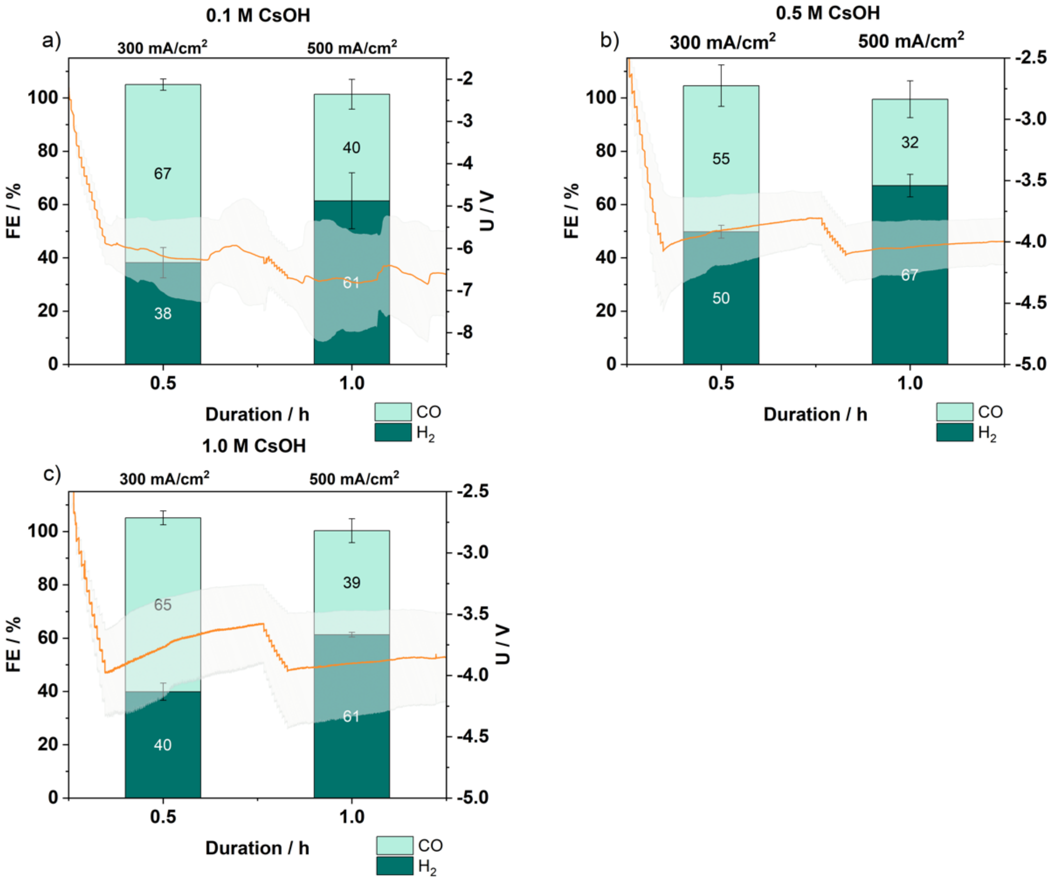

Figure S10. Detailed overview of the achieved FE_{CO} (dark green) and FE_{H2} (light green) pictured as bar chart with the corresponding cell voltage (orange line) achieved in 60 °C catalysis at 300 and 500 mA/cm^2 for 30 min each. The GDEs were coated with 0.5 mg/cm^2 of **Ag(dithiacyclam)** and SuperP. Electrolysis was performed with a) 0.1 M CsOH; b) 0.5 M CsOH and c) 1.0 M CsOH.

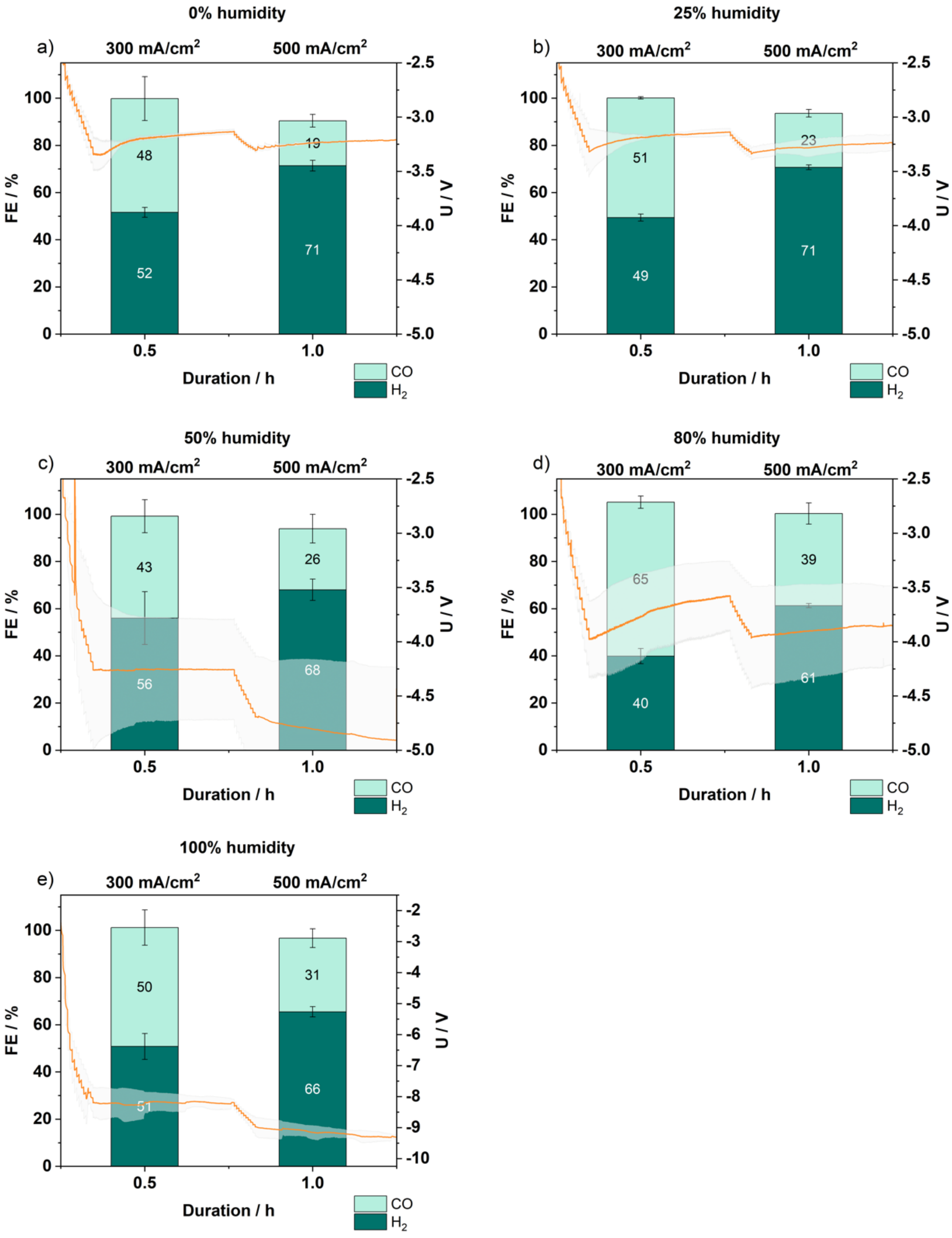

Figure S11. Detailed overview of the achieved FE_{CO} (dark green) and FE_{H2} (light green) pictured as bar chart with the corresponding cell voltage (orange line) achieved in 60 °C catalysis at 300 and 500 mA/cm^2 for 30 min each. The GDEs were coated with 0.5 mg/cm^2 of **Ag(dithiacyclam)** and SuperP. 1 M CsOH was used as anolyte. Electrolysis was performed with a relative humidity of a) 0%; b) 25% c) 50%; d) 80% and e) 100%.

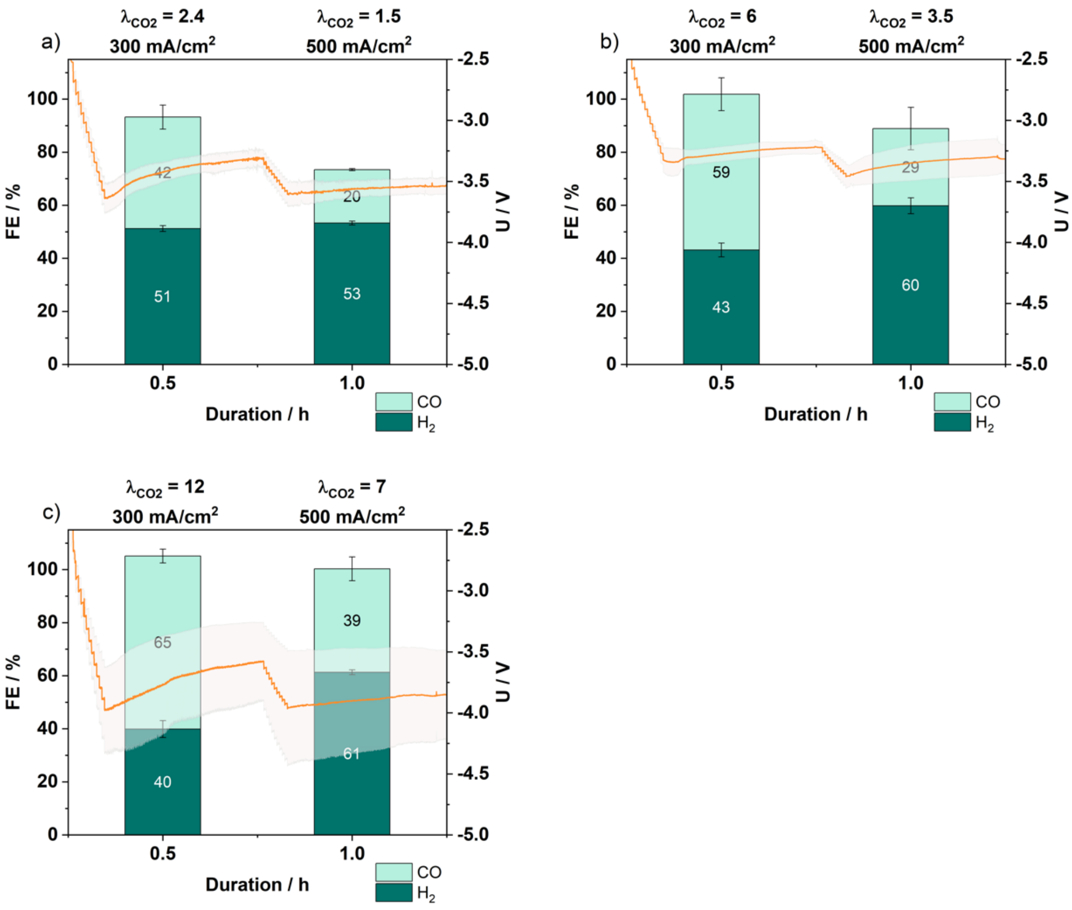

Figure S12. Detailed overview of the achieved FE_{CO} (dark green) and FE_{H2} (light green) pictured as bar chart with the corresponding cell voltage (orange line) achieved in 60 °C catalysis at 300 and 500 mA/cm^2 for 30 min each. The GDEs were coated with 0.5 mg/cm^2 of **Ag(dithiacyclam)** and SuperP. 1 M CsOH was used as anolyte. Electrolysis was performed with a CO_2 stream of a) 10 mL/min; b) 25 mL/min and c) 50 mL/min. The corresponding λ_{CO2} values are given in the graphs.

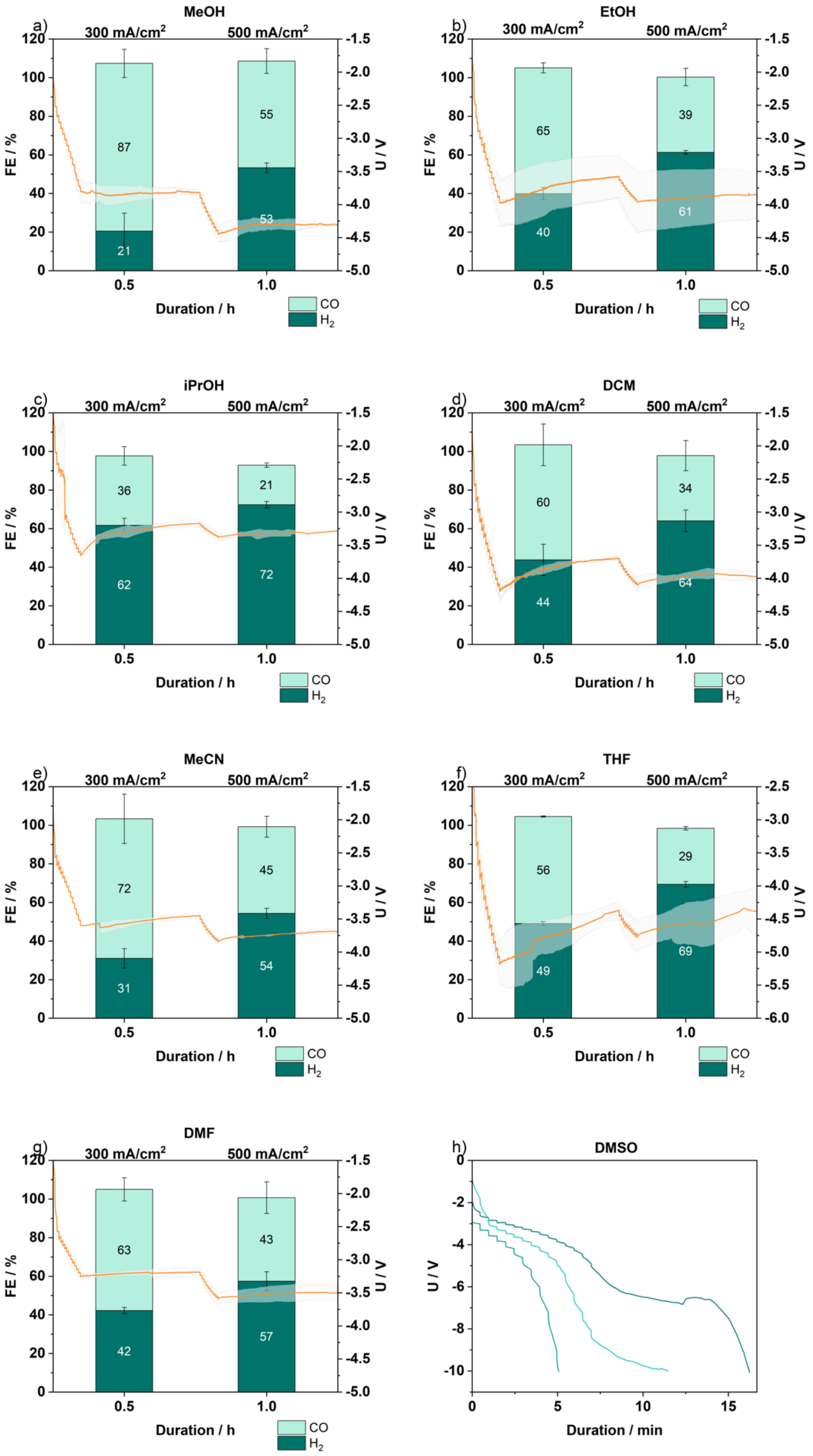

Figure S13. Detailed overview of the achieved FE_{CO} (dark green) and FE_{H2} (light green) pictured as bar chart with the corresponding cell voltage (orange line) achieved in 60 °C catalysis at 300 and 500 mA/cm² for 30 min each. The GDEs were coated with 0.5 mg/cm² of **Ag(dithiacyclam)** and SuperP. 1 M CsOH was used as analyte. Dispersions for GDE fabrication have been prepared with 2 mL of a) MeOH; b) EtOH; c) iPrOH; d) DCM; e) MeCN; f) THF; g) DMF and h) DMSO.

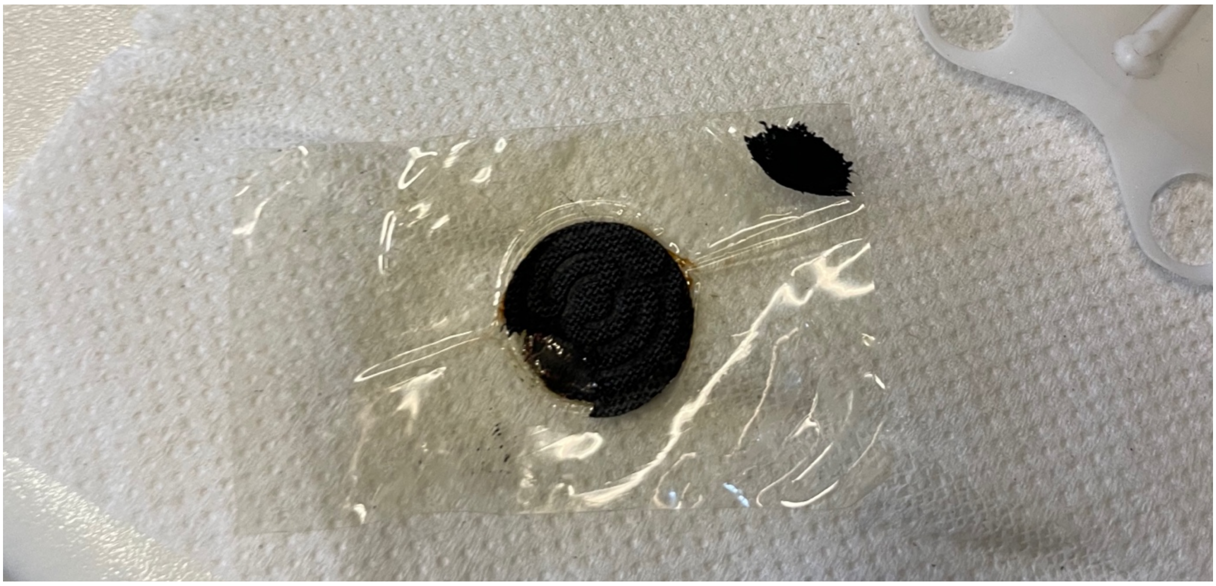

Figure S14. Image of a GDE prepared with DMSO which was „glued" to the membrane after an aborted electrolysis and needed to be ripped of to be separated.

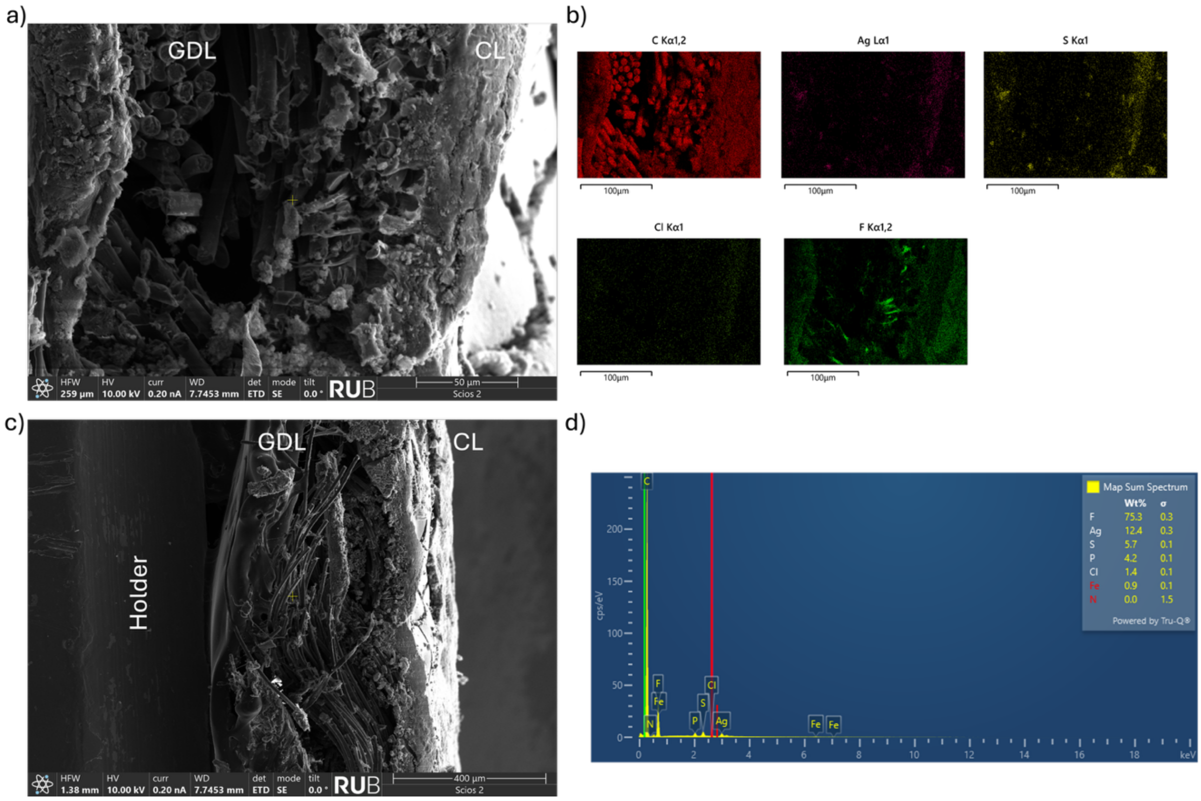

Figure S15. SEM/EDX images recorded of a pristine GDE prepared with iPrOH as dispersion solvent. SEM images recorded with a magnification of a) 800x and c) 150x; b) Elemental Mapping; d) EDX spectrum.

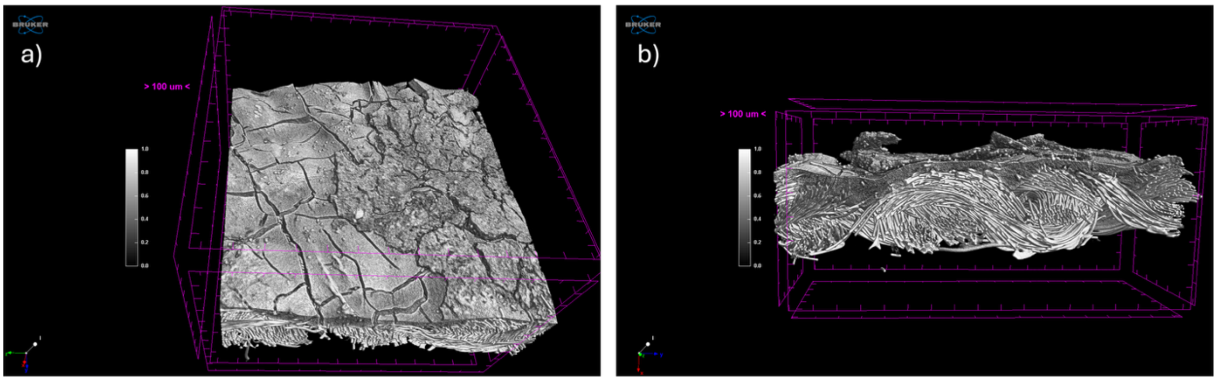

Figure S16. CT images of a pristine GDE prepared with iPrOH. a) Surface; b) cross section.

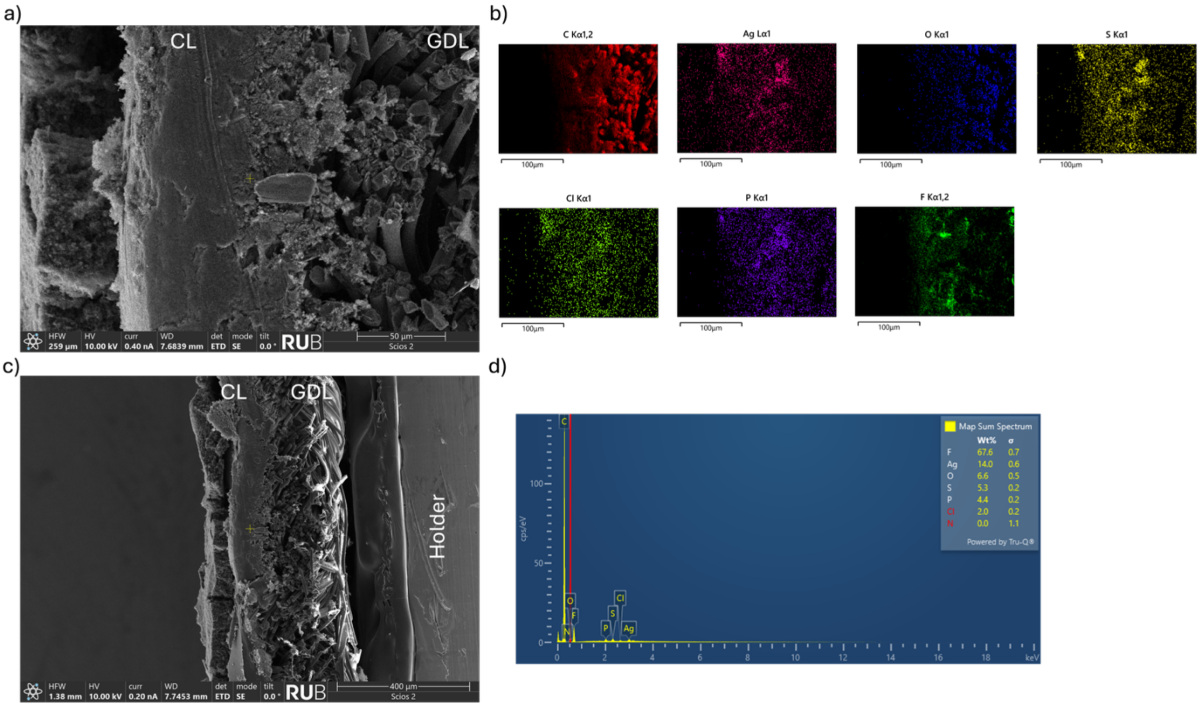

Figure S17. SEM/EDX images recorded of a pristine GDE prepared with EtOH as dispersion solvent. SEM images recorded with a magnification of a) 800x and c) 150x; b) Elemental Mapping; d) EDX spectrum.

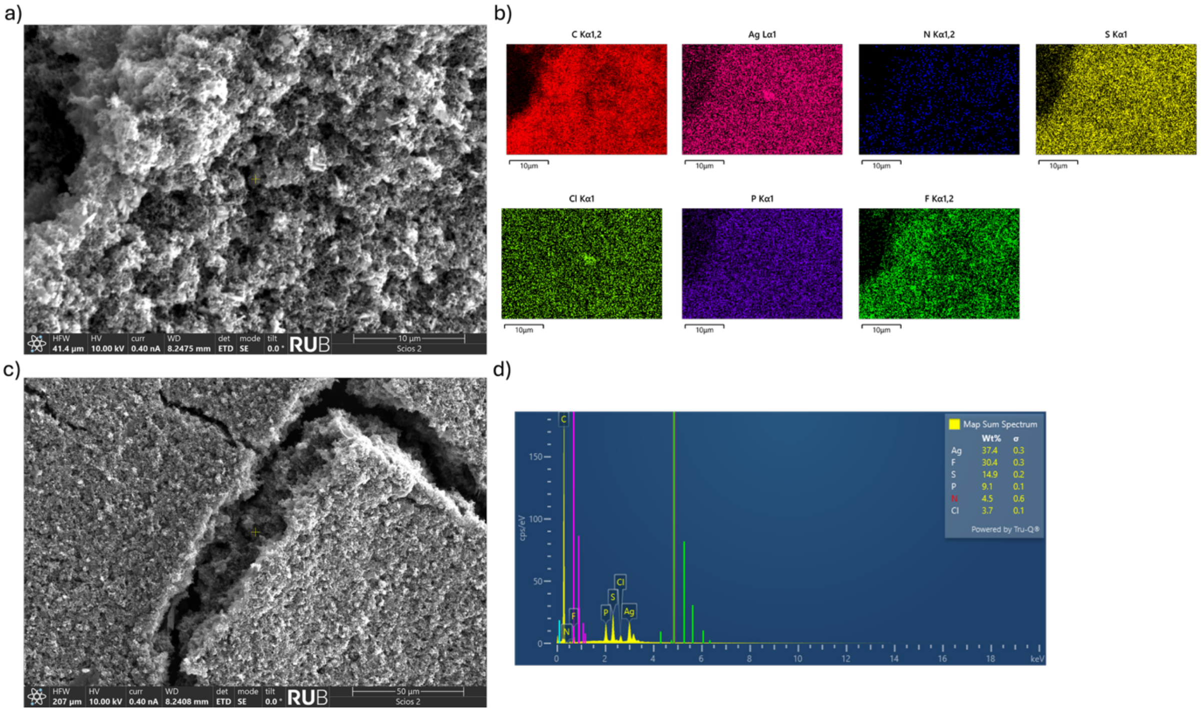

Figure S18. SEM/EDX images recorded of a prisitine GDE prepared with EtOH as dispersion solvent. SEM images recorded with a magnification of a) 5000x and c) 1000x; b) Elemental Mapping; d) EDX spectrum.

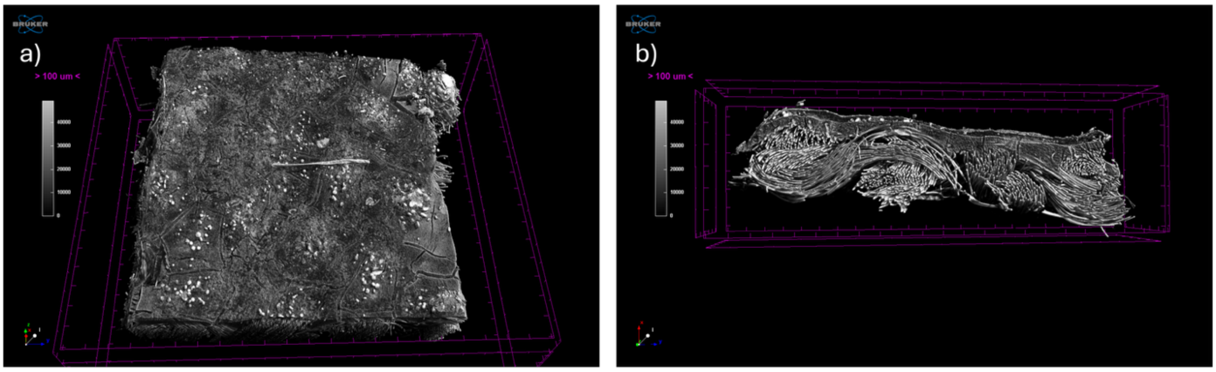

Figure S19. CT images of a pristine GDE prepared with EtOH. a) Surface; b) cross section.

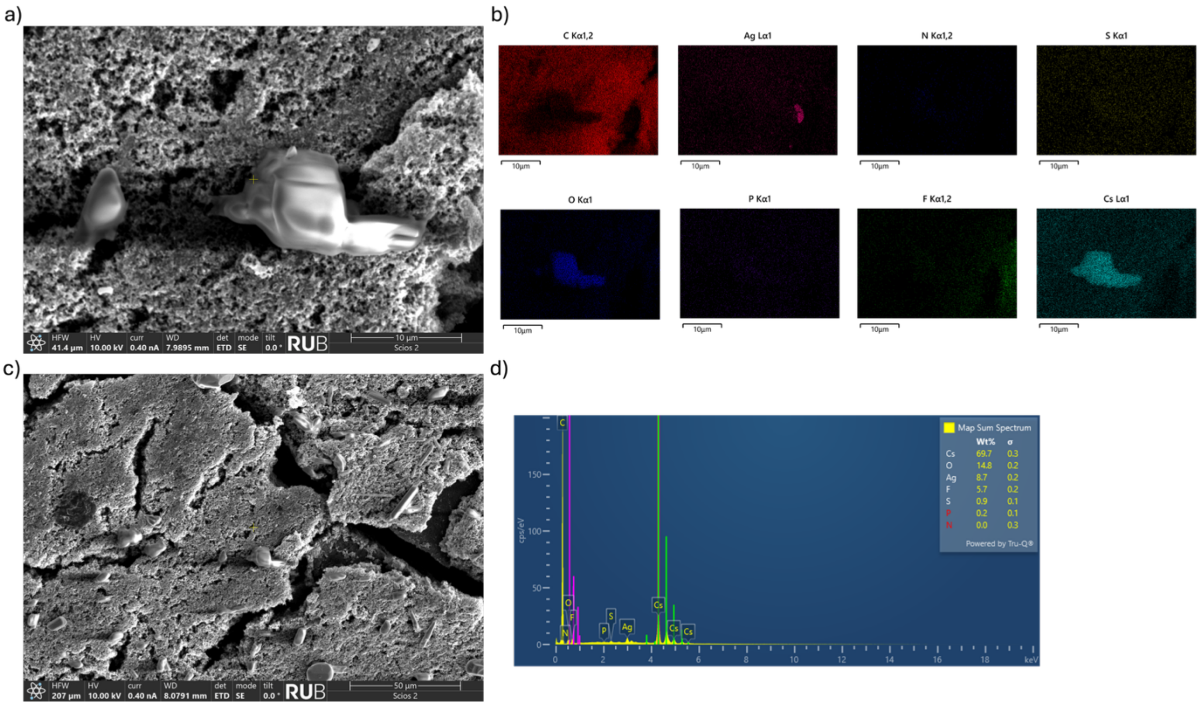

Figure S20. SEM/EDX images recorded of a GDE prepared with EtOH as dispersion solvent utilized in 60 °C catalysis. SEM images recorded with a magnification of a) 5000x and c) 1000x; b) Elemental Mapping; d) EDX spectrum.

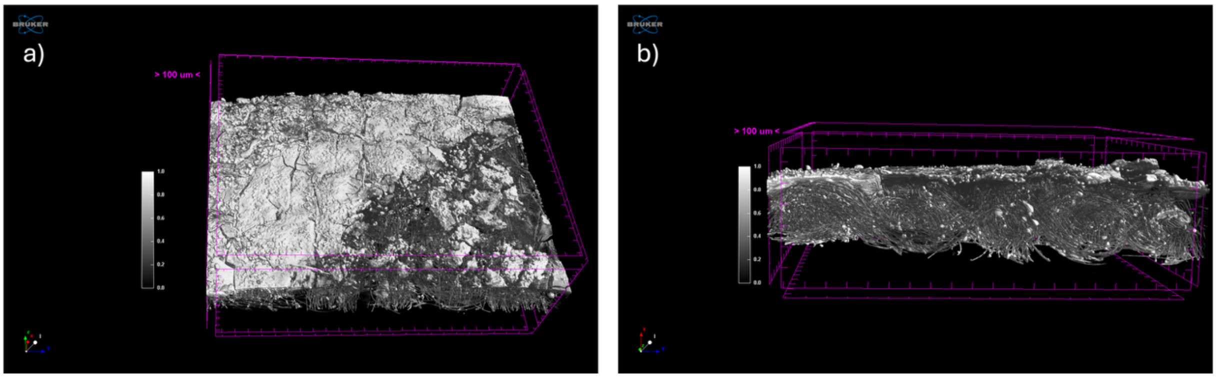

Figure S21. CT images of a utilized GDE prepared with EtOH. a) Surface; b) cross section.

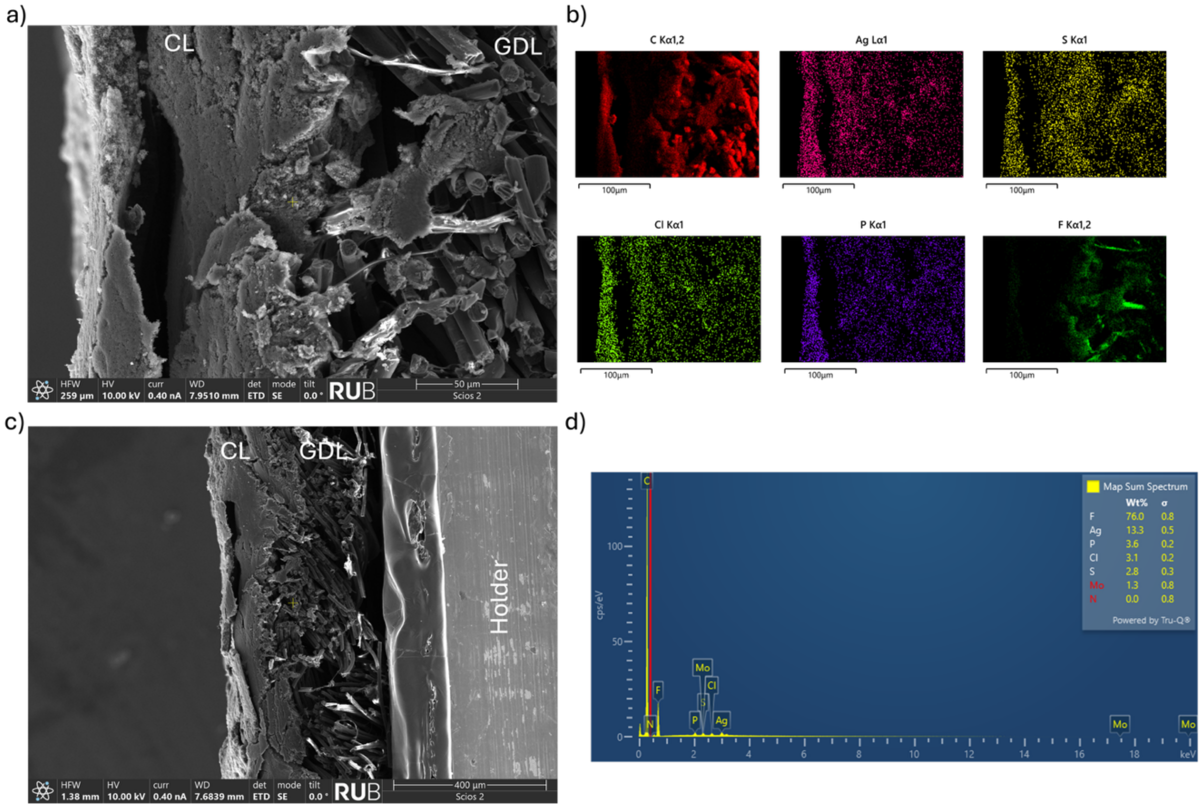

Figure S22. SEM/EDX images recorded of a pristine GDE prepared with MeOH as dispersion solvent. SEM images recorded with a magnification of a) 800x and c) 150x; b) Elemental Mapping; d) EDX spectrum.

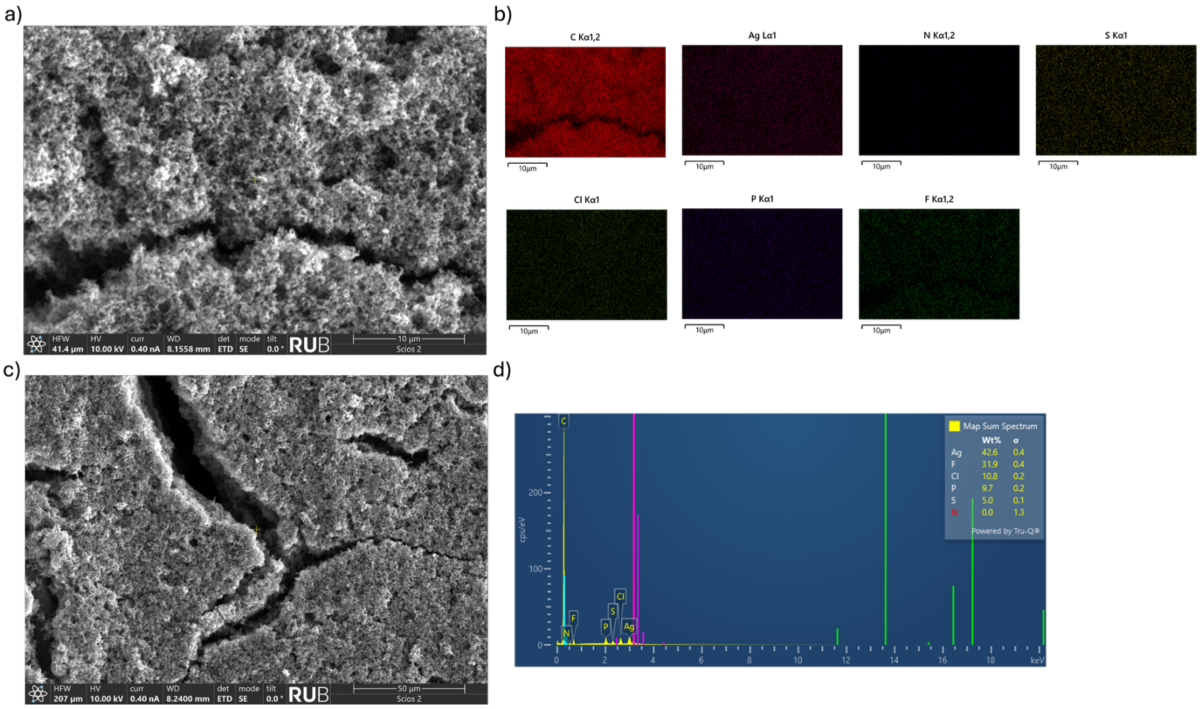

Figure S23. SEM/EDX images recorded of a prisitine GDE prepared with MeOH as dispersion solvent. SEM images recorded with a magnification of a) 5000x and c) 1000x; b) Elemental Mapping; d) EDX spectrum.

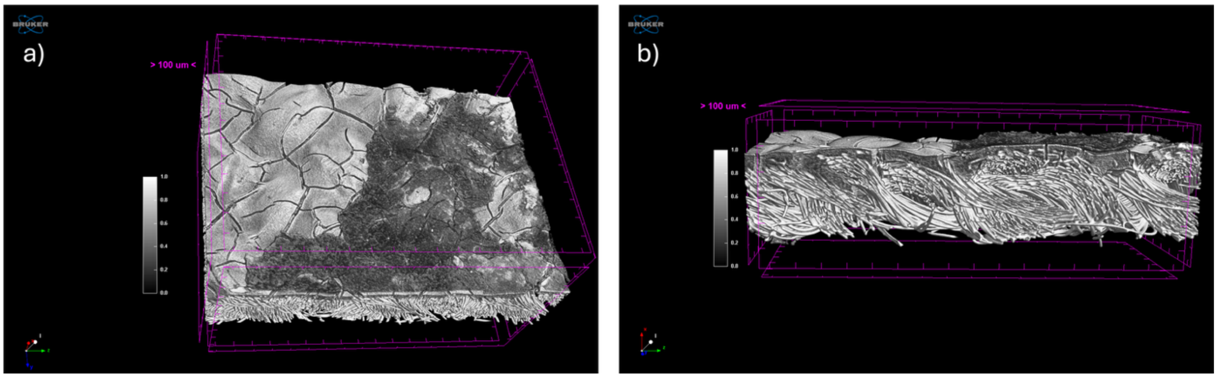

Figure S24. CT images of a pristine GDE prepared with MeOH. a) Surface; b) cross section.

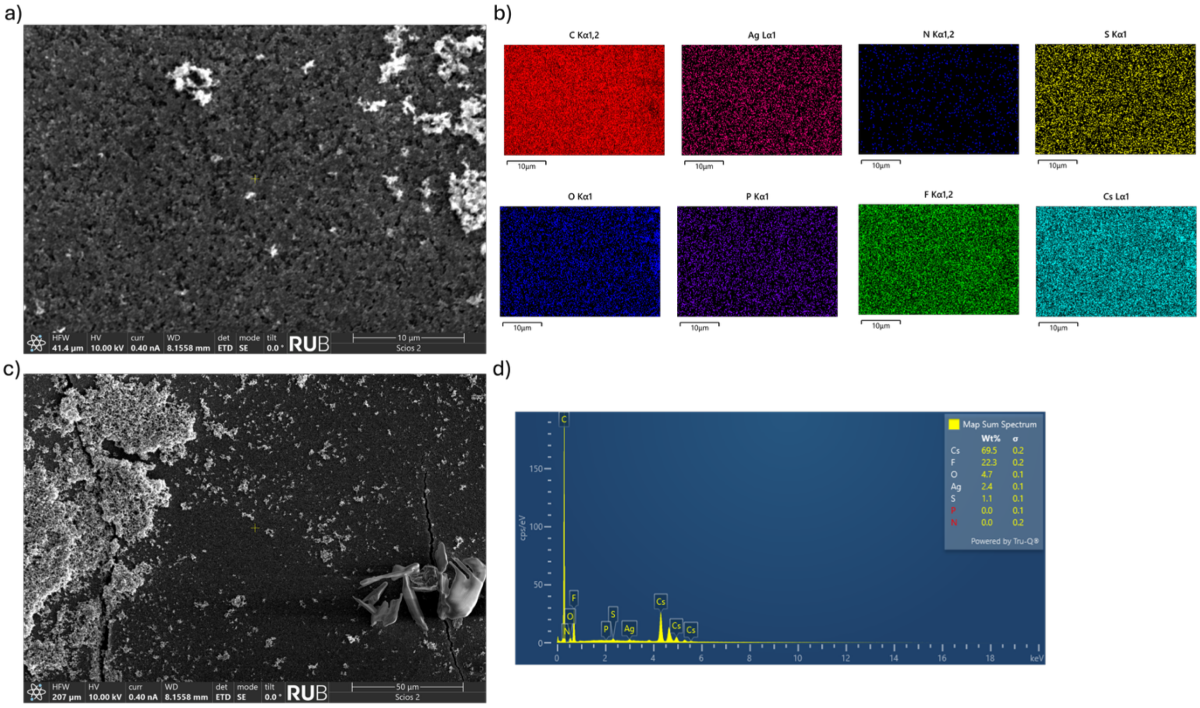

Figure S25. SEM/EDX images recorded of a GDE prepared with MeOH as dispersion solvent utilized in 60 °C catalysis. SEM images recorded with a magnification of a) 5000x and c) 1000x; b) Elemental Mapping; d) EDX spectrum.

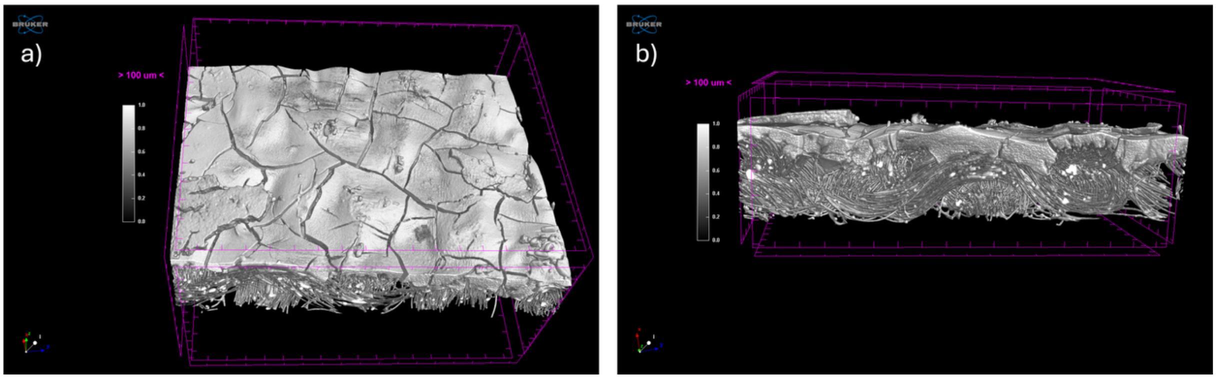

Figure S26. CT images of a utilized GDE prepared with MeOH. a) Surface; b) cross section.

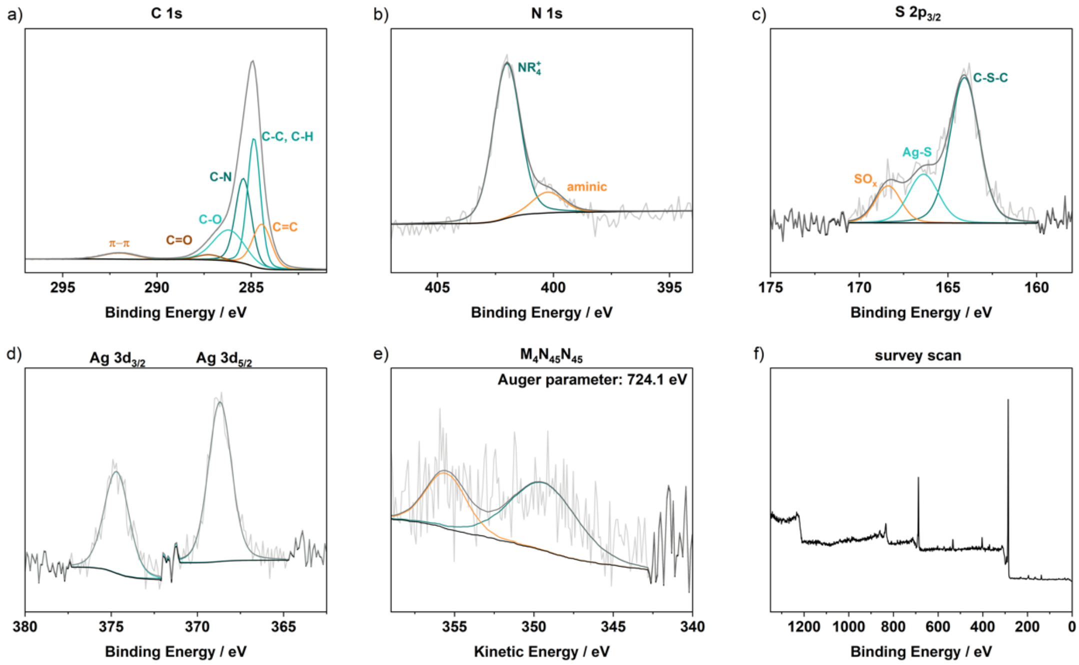

Figure S27. XPS spectra of a pristine GDE, prepared with MeOH as dispersion solvent, used for 60 °C electrolysis of the following orbitals a) C 1s; b) N 1s; c) S 2p; d) Ag 3d; e) Ag $M_4N_{45}N_{45}$ Auger peaks with the corresponding Auger parameter and f) survey scan.

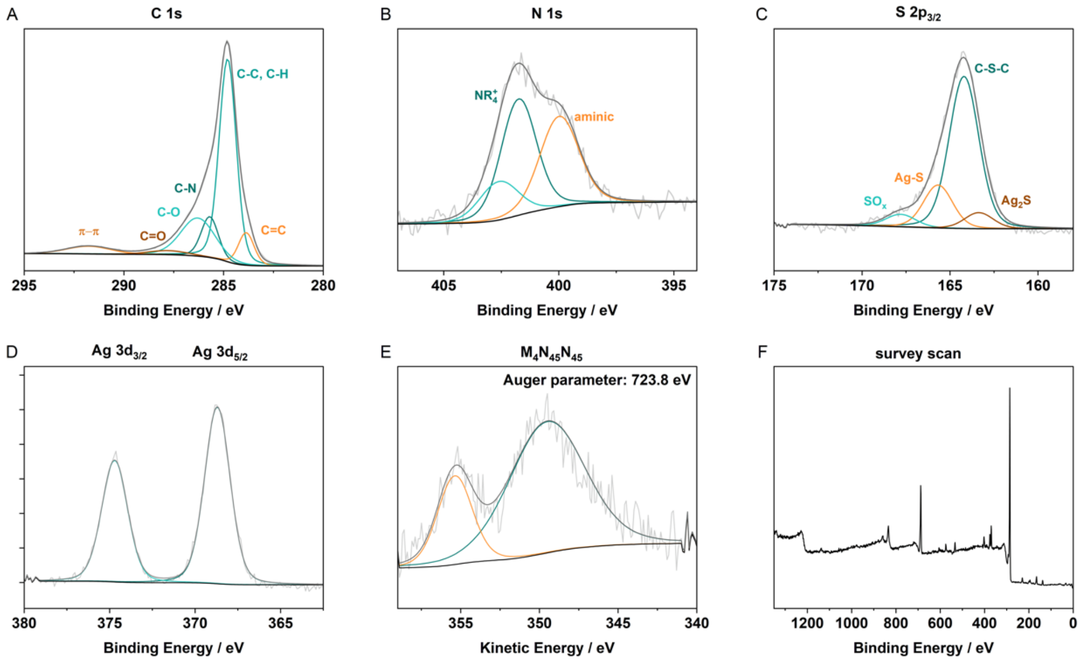

Figure S28. XPS spectra of a pristine GDE, prepared with EtOH as dispersion solvent, used for 60 °C electrolysis of the following orbitals a) C 1s; b) N 1s; c) S 2p; d) Ag 3d; e) Ag $M_4N_{45}N_{45}$ Auger peaks with the corresponding Auger parameter and f) survey scan.

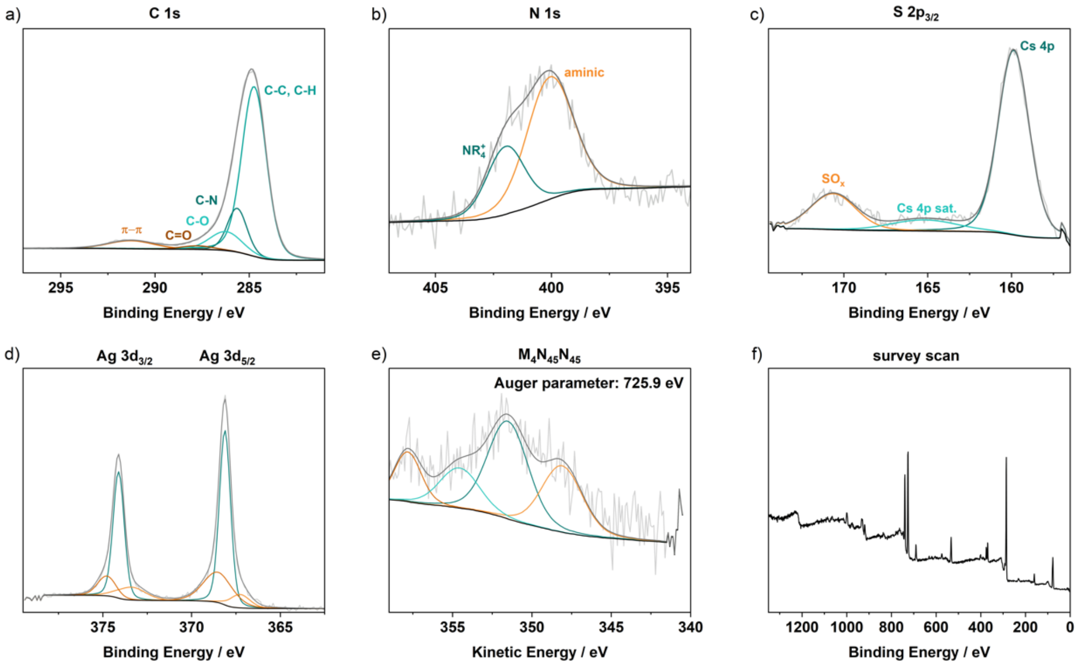

Figure S 29. XPS spectra of a utilized GDE, prepared with MeOH as dispersion solvent, applied in 60 °C electrolysis of the following orbitals a) C 1s; b) N 1s; c) S 2p; d) Ag 3d; e) Ag $M_4N_{45}N_{45}$ Auger peaks with the corresponding Auger parameter and f) survey scan.

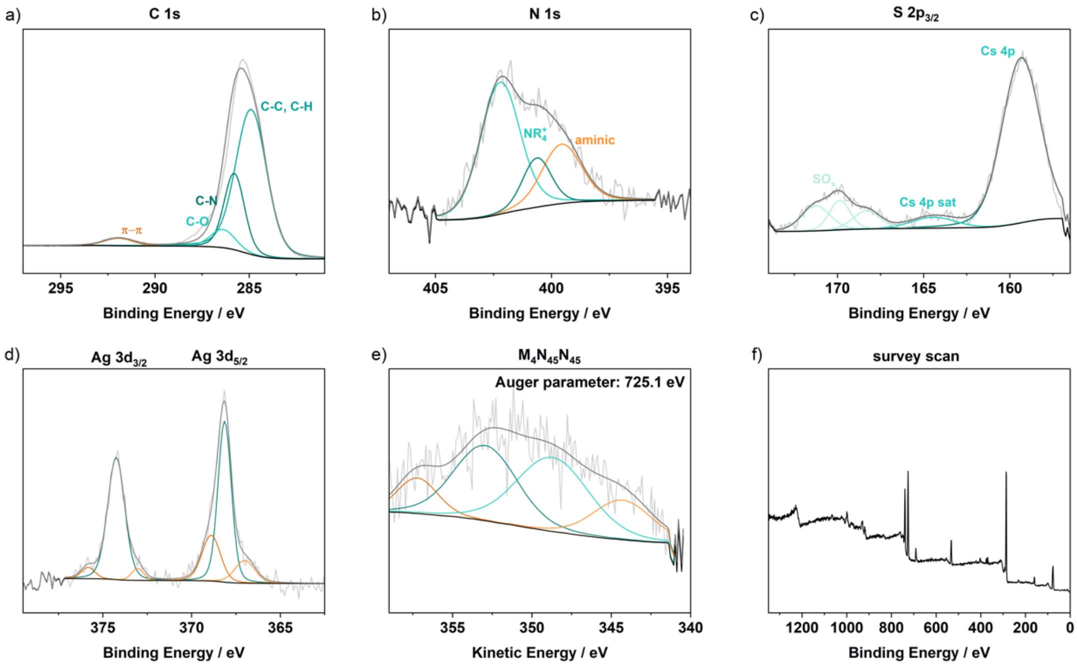

Figure S30. XPS spectra of a utilized GDE, prepared with EtOH as dispersion solvent, applied in 60 °C electrolysis of the following orbitals a) C 1s; b) N 1s; c) S 2p; d) Ag 3d; e) Ag $M_4N_{45}N_{45}$ Auger peaks with the corresponding Auger parameter and f) survey scan.

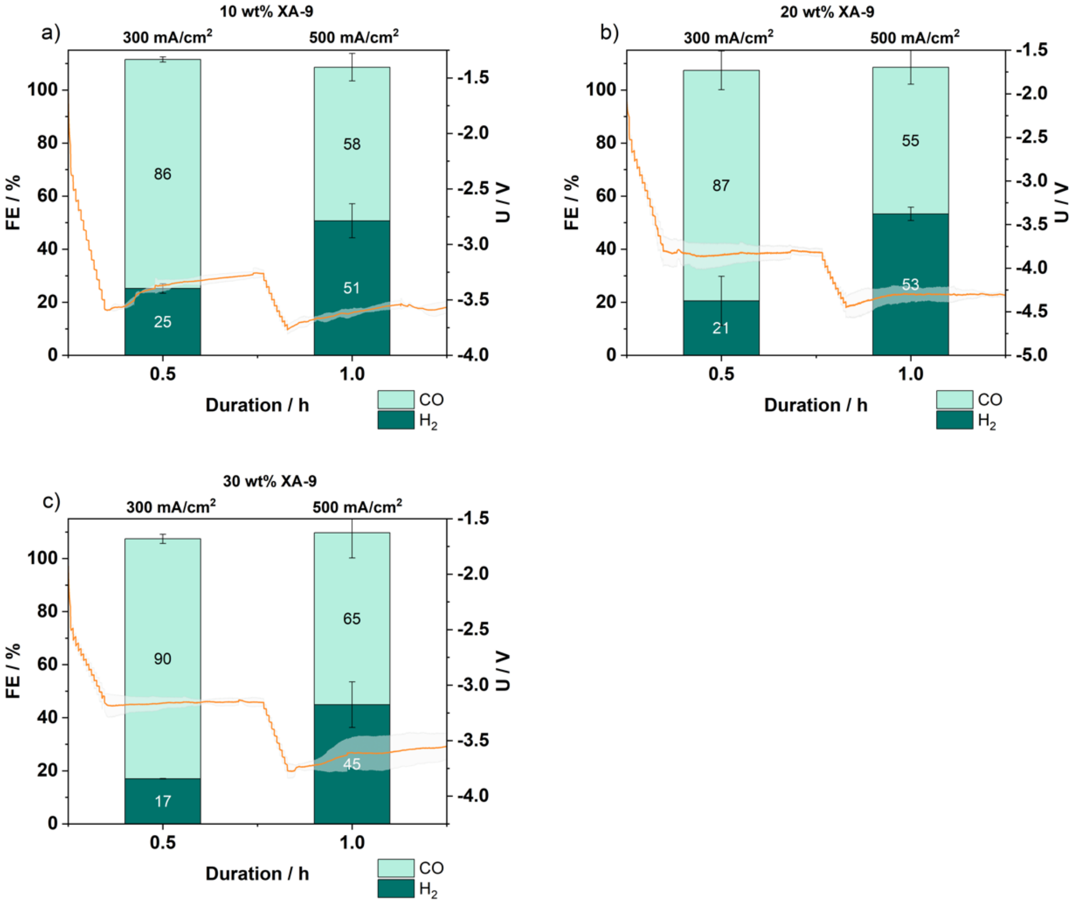

Figure S31. Detailed overview of the achieved FE_{CO} (dark green) and FE_{H2} (light green) pictured as bar chart with the corresponding cell voltage (orange line) achieved in 60 °C catalysis at 300 and 500 mA/cm^2 for 30 min each. The GDEs were coated with 0.5 mg/cm^2 of **Ag(dithiacyclam)** and SuperP. Dispersions for GDE fabrication have been prepared with 2 mL of MeOH and with a binder content of a) 10 wt%; b) 20 wt% and c) 30 wt%. 1 M CsOH was used as anolyte.

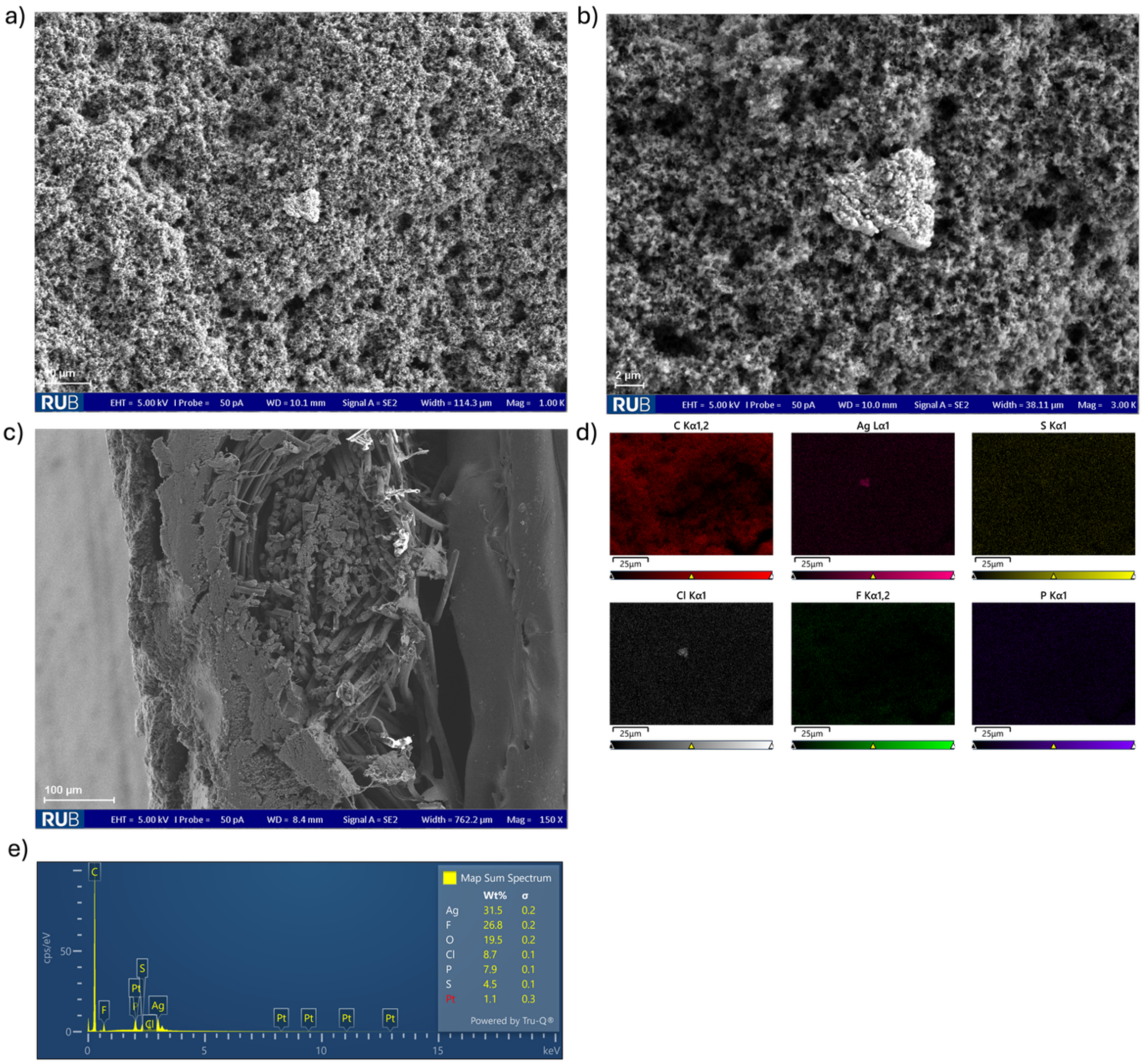

Figure S32. SEM/EDX images recorded of a prisitine GDE prepared with MeOH as dispersion solvent and 30 wt% SustainION XA-9 binder. SEM images recorded with a magnification of a) 1000x and b 3000x; c) crossectional SEM image at a magnification of 800x; d) Elemental Mapping; e) EDX spectrum.

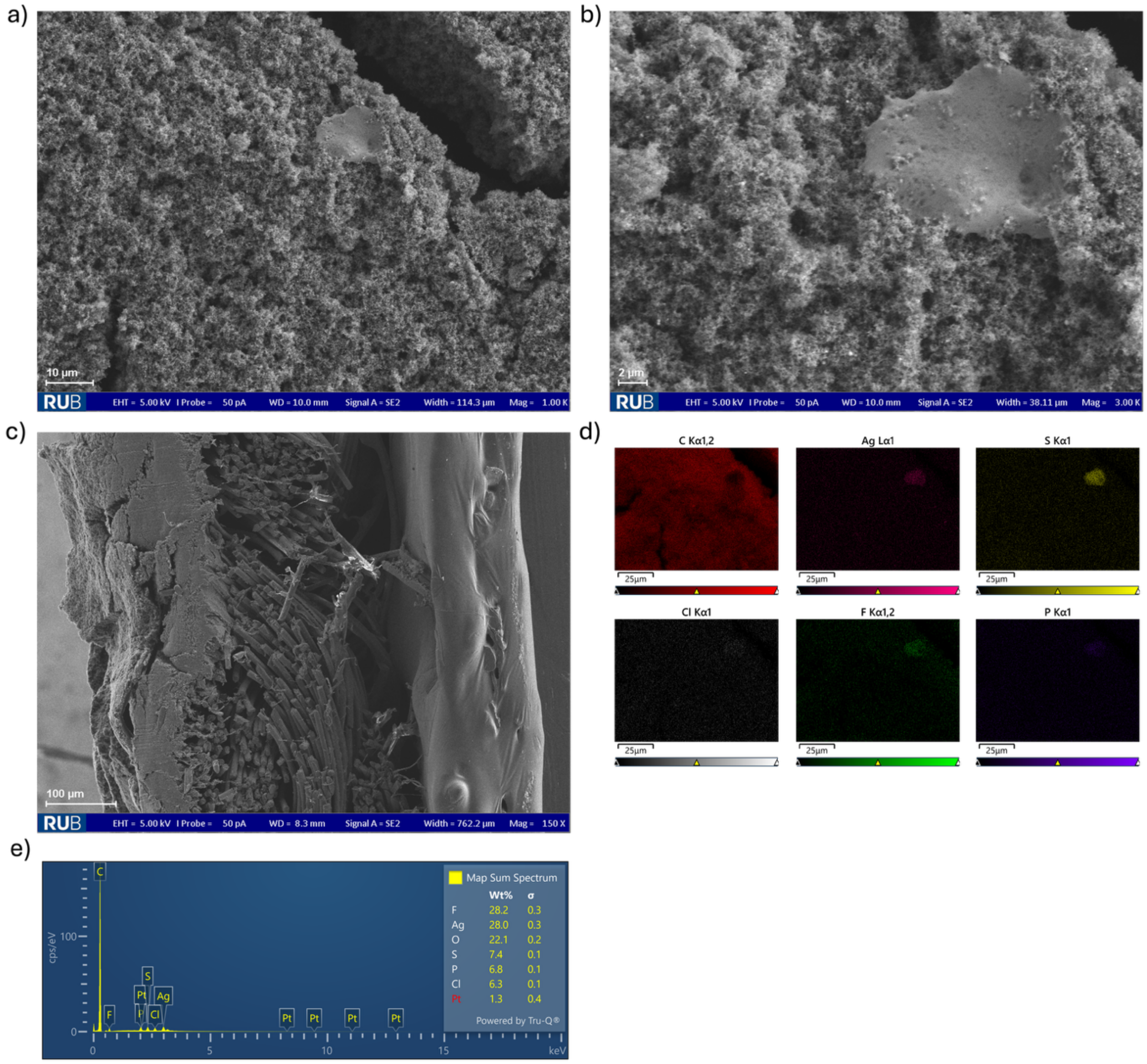

Figure S33. SEM/EDX images recorded of a prisitine GDE prepared with MeOH as dispersion solvent and 10 wt% SustainION XA-9 binder. SEM images recorded with a magnification of a) 1000x and b 3000x; c) crossectional SEM image at a magnification of 800x; d) Elemental Mapping; e) EDX spectrum.

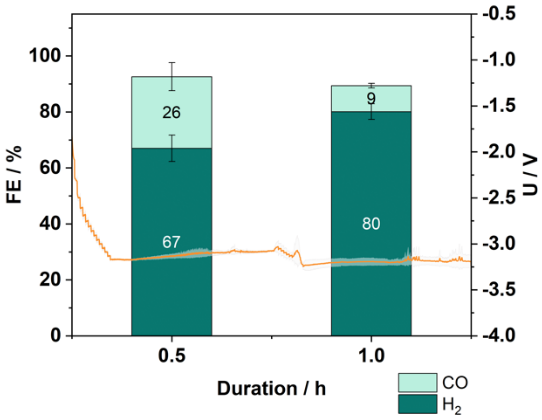

Figure S34. Detailed overview of the achieved FE_{CO} (dark green) and FE_{H2} (light green) pictured as bar chart with the corresponding cell voltage (orange line) achieved in 60 °C catalysis at 300 and 500 mA/cm² for 30 min each. The GDEs were coated with 0.5 mg/cm² of SuperP as carbon support and 0.22 mg/cm² Ag NPs (<40 nm) and 33 wt% binder. 1 M CsOH was used as anolyte.

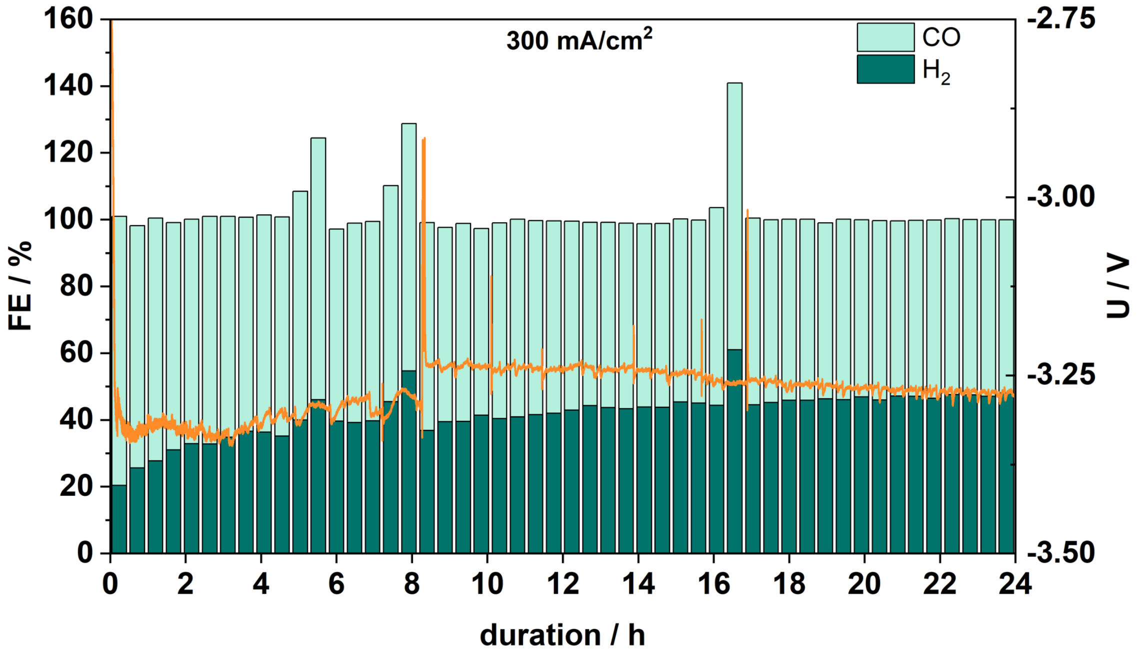

Figure S35. Detailed overview of the achieved FE_{CO} (dark green) and FE_{H2} (light green) pictured as bar chart with the corresponding cell voltage (orange line) achieved in 60 °C catalysis at 300 mA/cm^2 for 24 h. Product gas analysis was performed every 30 min. The GDEs were coated with 0.5 mg/cm^2 of **Ag(dithiacyclam)** and SuperP. Dispersions for GDE fabrication have been prepared with 2 mL of MeOH and with a binder content of 30 wt%. 1 M CsOH was used as analyte.

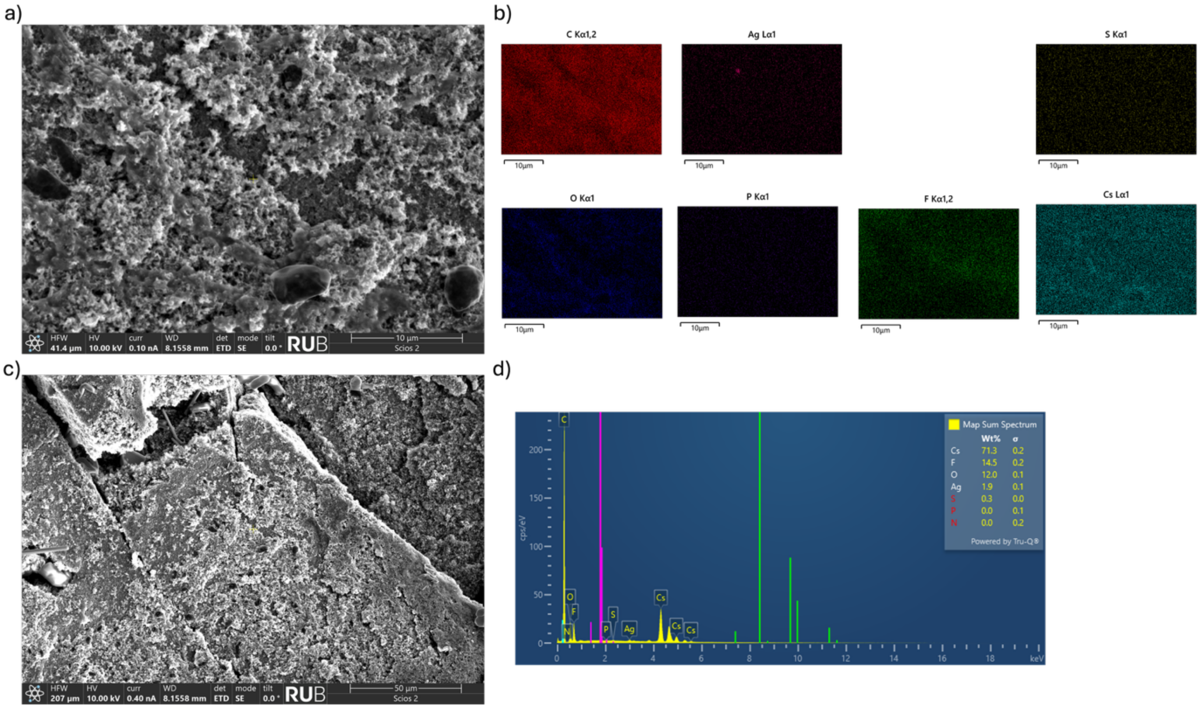

Figure S36. SEM/EDX images recorded of a GDE prepared with MeOH as dispersion solvent utilized in 60 °C catalysis for 24 h. SEM images recorded with a magnification of a) 5000x and c) 1000x; b) Elemental Mapping; d) EDX spectrum.

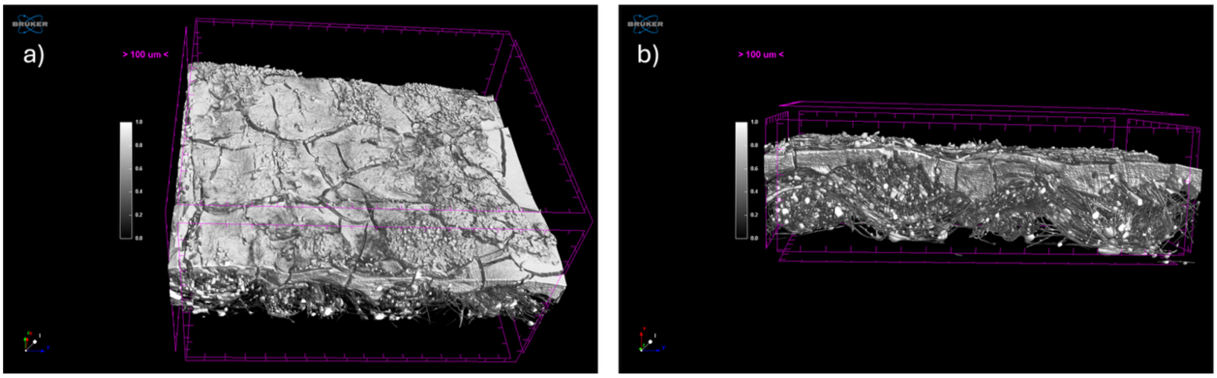

Figure S37. CT images of a GDE applied in 24 h electrolysis prepared with MeOH. a) Surface; b) cross section.

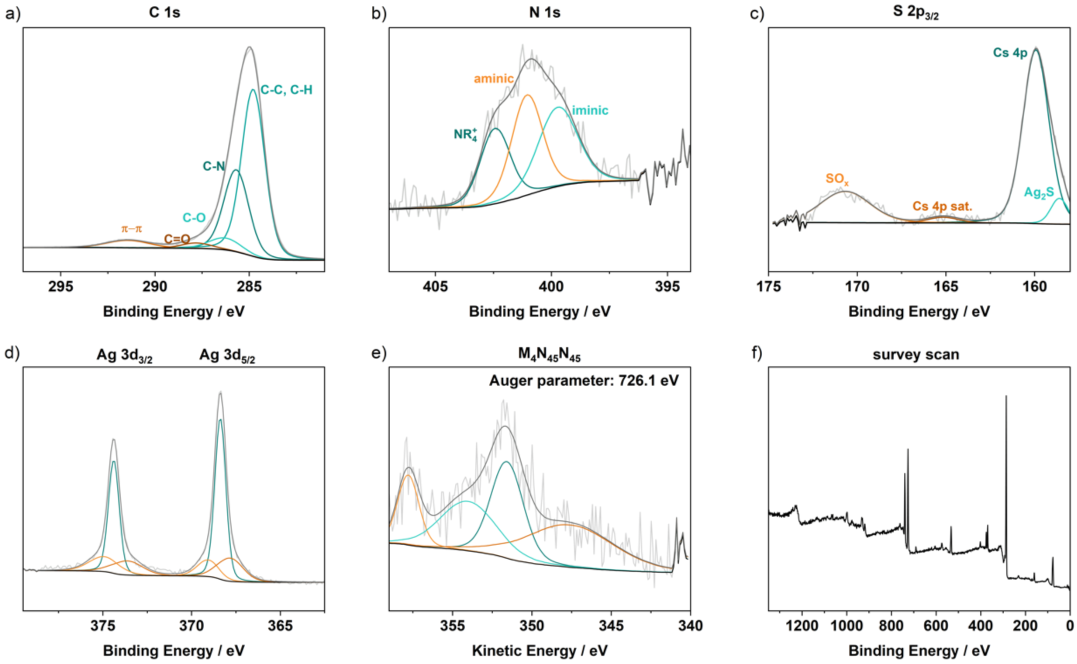

Figure S38. XPS spectra of a utilized GDE, prepared with EtOH as dispersion solvent, applied in 60 °C electrolysis of the following orbitals a) C 1s; b) N 1s; c) S 2p; d) Ag 3d; e) Ag $M_4N_{45}N_{45}$ Auger peaks with the corresponding Auger parameter and f) survey scan.

Additional tables

Table S1. Display oft he λ_{CO2} value depending on the CO_2 flow rate and the applied current densities.

CO_2 Flow / mL/min	λ_{CO2}	
	300 mA/cm^2	500 mA/cm^2
10	2.4	1.5
25	6	3.5
50	12	7

Table S2. Tabular overview comparing the performance of Ag(dithiacyclam) with reported silver-based and molecular systems in ZGEs with respect to applied current density and achieved FE_{CO} and cell voltage.

Catalyst	*Applied current density / mA/cm²*	*FE_{CO} / %*	*Cell voltage / V*	*Reference*
Ag(dithiacyclam)	50	97	2.7	**This work**
Ag(dithiacyclam)	300	90	3.2	**This work**
Ag(dithiacyclam)	500	65	3.6	**This work**
Ag(BIAN-OC₆)	100	66	2.9	[4]
Ag(BIAN-OC₆)	300	92	3.1	[4]
Ag(BIAN-OC₆)	600	66	3.6	[4]
AgTPP	50	99	2.6	[5]
AgTPP	100	93	3.0	[5]
CoPc	150	95	2.5	[6]
Co(TPPoCE2)	300	43	3.3	[7]
NiCe(salen)	75	82	2.5	[8]
Ag-CP	300	96	3.1	[9]
Ag/C_{SuperP}	300	80	3.5	[10]
Ag_{Coral}	100	95	2.75	[11]
$Ag/C_{anchored}$	500	90	2.9	[12]

References

1 P. Gerschel, K. Warm, E. R. Farquhar, U. Englert, M. L. Reback, D. Siegmund, K. Ray and U.-P. Apfel, *Dalton Trans.*, 2019, **48**, 5923–5932.

2 A. Johnson, L. Iffland, K. Singh, U.-P. Apfel and K. Suntharalingam, *Dalton Trans.*, 2021, **50**, 5779–5783.

3 L. Hoof, N. Thissen, K. Pellumbi, K. Junge Puring, D. Siegmund, A. K. Mechler and U.-P. Apfel, *Cell Reports Physical Science*, 2022, **3**, 100825.

4 K. Pellumbi, D. Krisch, C. Rettenmaier, H. Awada, H. Sun, L. Song, S. A. Sanden, L. Hoof, L. Messing, K. J. Puring, D. Siegmund, B. R. Cuenya, W. Schöfberger and U.-P. Apfel, *Cell Reports Physical Science*, 2023, **4**, 101746.

5 W. Wiesner, J. Y. M. Arias, J. Jökel, R. Cao and U.-P. Apfel, *Chem. Commun.*, 2024, **60**, 14668–14671.

6 S. Ren, D. Joulié, D. Salvatore, K. Torbensen, M. Wang, M. Robert and C. P. Berlinguette, *Science*, 2019, **365**, 367–369.

7 W. Wiesner, C. Wilhelm, R. C. Hoffmann, P. Stahl, K. Pellumbi, J. Jökel, I. Ivanović-Burmazović and U.-P. Apfel, *Angew Chem Int Ed*, 2026, e25189.

8 F. Yari, A. Aljabour, H. Awada, J. Michalke, N. Kumari, H. Coskun-Aljabour, S. Roy, D. Krisch and W. Schöfberger, *ACS Appl. Energy Mater.*, 2024, **7**, 10052–10060.

9 R. Wang, H. Haspel, A. Pustovarenko, A. Dikhtiarenko, A. Russkikh, G. Shterk, D. Osadchii, S. Ould-Chikh, M. Ma, W. A. Smith, K. Takanabe, F. Kapteijn and J. Gascon, *ACS Energy Lett.*, 2019, **4**, 2024–2031.

10 K. Seteiz, J. N. Häberlein, P. A. Heizmann, J. Disch and S. Vierrath, *RSC Adv.*, 2023, **13**, 18916–18926.

11 W. H. Lee, Y.-J. Ko, Y. Choi, S. Y. Lee, C. H. Choi, Y. J. Hwang, B. K. Min, P. Strasser and H.-S. Oh, *Nano Energy*, 2020, **76**, 105030.

12 K. Seteiz, H. Grammel, J. N. Häberlein, P. A. Heizmann, L. Metzler, D. Rusitov, M. Günthel, M. Knäbbeler-Buß, S. Vierrath and J. Disch, *Nano Energy*, 2025, **134**, 110597.
